# Elevated non-invasive liver fibrosis scores at admission are independent risk factors for severe COVID-19: a retrospective cohort study from 2020 to 2024

**DOI:** 10.3389/fmed.2025.1727318

**Published:** 2026-01-05

**Authors:** Lucia Cabrejos Hirashima, Nicole E. Naiman, Amyn A. Malik, Mamta K. Jain

**Affiliations:** 1 Department of Internal Medicine, University of Texas Southwestern Medical Center, Dallas, TX, United States; 2 Department of Epidemiology, O’Donnell School of Public Health, University of Texas Southwestern Medical Center, Dallas, TX, United States; 3 Parkland Hospital System, Dallas, TX, United States

**Keywords:** SARS-CoV-2, COVID-19, aspartate aminotransferase to platelet ratio index, fibrosis-4 index, non-alcoholic fatty liver disease fibrosis score, non-invasive liver fibrosis score

## Abstract

**Background:**

COVID-19 patients frequently present with abnormal liver function tests (LFTs) and elevated non-invasive liver fibrosis scores, such as the fibrosis-4 index (FIB-4), the non-alcoholic fatty liver disease fibrosis score (NFS), and the aspartate aminotransferase (AST) to platelet ratio index (APRI). While elevated LFTs and non-invasive liver fibrosis scores in COVID-19 patients have been associated with poor COVID-19 outcome, most of those data were collected before the dominance of the Omicron variant and shift in disease presentation to a milder respiratory presentation.

**Methods:**

This was a retrospective cohort study of 4,565 non-pregnant adults admitted with COVID-19 from 03/01/2020 to 12/31/2024. We examined the association of LFT and non-invasive liver fibrosis score derangements near admission with relative risk of severe COVID-19, a composite outcome defined as death and/or requirement of organ support. Subgroup analyses included: a “non-liver disease subgroup” (patients without known prior liver disease, viral hepatitis, or prior remdesivir use), a “room air subgroup” (patients who remained on room air during the first 24 h of admission), and viral variant subgroups defined by date. Multivariable regression models were compared via area under the receiver operating characteristic (ROC) curve and Akaike Information Criterion (AIC).

**Results:**

Elevations in FIB-4, NFS, and APRI were associated with increased risk of severe COVID-19 in the total cohort and across various subgroups. High FIB-4 (>2.67) and intermediate APRI (0.5–1.0) were associated with increased risk of severe COVID-19 in the total cohort (FIB-4 RR: 2.25, 95% CI 1.81–2.79; APRI RR: 1.53, 95% CI 1.32–1.79), with similar results in the non-liver disease subgroup and across the Pre-Delta, Delta, and Omicron subgroups. High NFS (>0.675) was associated with increased risk of severe COVID-19 in the total cohort (RR: 2.33, 95% CI 1.83–2.97), with similar results in the room air, Pre-Delta, and Delta subgroups. Overall, the models had similar outcome discrimination based on area under the ROCs, but the FIB-4 models had the best fit based on AICs.

**Conclusion:**

Elevated non-invasive liver fibrosis scores at admission were associated with risk of severe COVID-19 across variants regardless of the baseline respiratory status or liver health of COVID-19 patients in this cohort.

## 1 Introduction

Coronavirus disease 2019 (COVID-19) emerged as a predominantly respiratory illness, with mortality often attributed to complications such as acute respiratory distress (1). As new data became available, extrapulmonary manifestations of COVID-19, including effects on the hepatobiliary system were documented (2–5).

Liver function test (LFT) abnormalities, especially elevated transaminases, are frequently reported among COVID-19 patients (5–7). Prior studies have linked LFT abnormalities with poor COVID-19 outcomes such as severe disease, intensive care unit (ICU) admission, mechanical ventilation, and mortality (5, 8).

Certain metrics calculated based on LFTs as well as other laboratory values and/or characteristics will be referred to as composite liver scores for the purposes of this study. Composite liver scores discussed here include the De Ritis ratio, liver pattern as determined by the R index (i.e., hepatocellular, cholestatic, or mixed), and three non-invasive liver fibrosis scores: the fibrosis-4 index (FIB-4), the non-alcoholic fatty liver disease fibrosis score (NFS), and the aspartate aminotransferase (AST) to platelet ratio index (APRI)(9–13). The non-invasive liver fibrosis scores (FIB-4, NFS, and APRI) are a subset of composite liver scores originally developed to estimate the likelihood and degree of fibrosis in patients with liver disease (11–13). The aforementioned composite liver scores have been shown to be associated with COVID-19 disease progression and poor outcome, with FIB-4 being the most widely studied in the context of COVID-19 (14–26). However, much of the available data are from early pandemic cohorts prior to the development and dominance of the Omicron variant and its subvariants (14, 16–18, 21–25).

Clinical presentations and disease severity have evolved with emerging variants (26–33). Some studies have investigated individual LFT abnormalities (i.e., abnormal AST, abnormal alanine aminotransferase (ALT), abnormal alkaline phosphatase (AP), abnormal bilirubin, etc.) across SARS-CoV-2 variants (19, 20, 34–38). However, few studies have examined the association of composite liver scores with COVID-19 outcome throughout the different SARS-CoV-2 variant-predominant periods (19, 20, 26). Of the aforementioned studies examining LFTs and/or composite liver scores across the evolution of COVID-19, only two were conducted in the United States (19, 26).

Given the widespread availability of LFTs and ease of calculating various composite liver scores in clinical settings, it is important to evaluate their association with patient outcomes throughout the evolution of COVID-19. Despite lower COVID-19 mortality rates compared to early pandemic levels, severe COVID-19 leading to hospitalization and death still persist (31, 32). In this retrospective cohort study, we examined the association between abnormal LFTs or composite liver scores at admission with severe COVID-19 in a safety-net hospital system in Dallas, Texas. This study aims to evaluate the use of LFTs and composite liver scores to prospectively predict severe COVID outcomes across patients with different variants and disease presentations. Our results can aid clinical practice by informing risk-stratification for patients who may require a higher level of care early on, even in the current COVID-19 era.

## 2 Materials and methods

### 2.1 Study population

This retrospective cohort study was performed with electronic medical record data from Parkland Memorial Hospital in Dallas, Texas from March 1, 2020, to December 31, 2024. Adults ≥ 18 years old admitted with COVID-19 were included in this study. COVID-19 was defined as a positive SARS-CoV-2 reverse transcriptase polymerase chain reaction (PCR) or antigen test within 2 weeks prior to and through the first 24 h of admission. Pregnant women, outpatients, and those discharged from the emergency department were not included in this study. We analyzed the first COVID-19 admission available per patient.

The primary outcome was severe COVID-19 defined by end-organ failure (indicated by requirement of organ support) and/or mortality associated with the COVID-19 admission. For the purposes of this study, end-organ failure was defined as the use of mechanical ventilation, intravenous vasopressors or inotropes, extracorporeal membrane oxygenation (ECMO) or continuous renal replacement therapy (CRRT; not including routine dialysis) during the COVID-19 admission. Mortality associated with the COVID-19 admission was defined by a date of death in the electronic medical record from any time during the admission through 7 days after the discharge date. Patients were considered to have severe COVID-19 at the time of admission and thus were excluded from the study if they met one or more of the following criteria: (1) required invasive mechanical ventilation within the first 24 h of admission; (2) required intravenous vasopressors (dopamine, vasopressin, phenylephrine, norepinephrine, or epinephrine) or inotropes (milrinone, dobutamine) on the day of or prior to admission. ECMO and CRRT start dates and times were not available.

### 2.2 Ethics and approvals

This study was approved, with a waiver of consent, by the University of Texas Southwestern Medical Center Institutional Review Board. This study met the STROBE statement criteria for cohort studies (Supplementary STROBE statement file) (39).

### 2.3 Data collection

Demographic information comprising age, sex, race, and ethnicity were collected alongside pre-existing comorbidities defined by ICD-10 codes, including hypertension, cardiovascular complications, respiratory disease, liver disease, viral hepatitis, chronic kidney disease (CKD), diabetes mellitus (DM), rheumatological disease, HIV infection, malignancy, paralytic syndromes, and dementia (Supplementary Table S1). Laboratory results before admission through the date of admission were used to identify additional cases of HIV, viral hepatitis, and DM.

Additional variables utilized in our analysis included body mass index (BMI), the highest oxygen requirement within the first 24 h of admission (hereafter referred to as baseline level of respiratory support), and remdesivir administration at least 1 day prior to laboratory sampling (hereafter referred to as prior remdesivir use; remdesivir start times to the hour were not available). BMI < 10 (kg/m^2^) or >100 (kg/m^2^) were assumed to be improperly entered into the medical record and considered missing (*N* = 42).

Baseline laboratory results were analyzed and defined as the first available results from the time of admission +/− 24 h, allowing for the inclusion of laboratory tests collected in the emergency department for patients who would then become admitted. Baseline aspartate aminotransferase (AST; U/L), alanine aminotransferase (ALT; U/L), alkaline phosphatase (AP; U/L), total bilirubin (mg/dL), direct bilirubin (mg/dL), gamma-glutamyl transferase (GGT, U/L), creatinine (mg/dL), C-reactive protein (CRP, mg/dL), D-dimer (mcg/mL), albumin (g/dL), hemoglobin (g/dL), lactate dehydrogenase (LDH; U/L), international normalized ratio (INR), prothrombin time (PT; seconds), platelet count (x10^9^/L), and blood urea nitrogen (BUN; mg/dL) results were analyzed. Some patients had multiple results at the same timepoint for the same laboratory metric; therefore, the mean value at that timepoint was used for analysis. Definitions of normal LFTs were as follows: AST ≤ 40 U/L, ALT ≤40 U/L, AP ≤ 130 U/L, total bilirubin ≤ 1.2 mg/dL, direct bilirubin ≤0.3 mg/dL, and GGT ≤ 61 U/L for males or ≤36 U/L for females. AP, total bilirubin, direct bilirubin, and GGT were classified as either normal or elevated. Definitions of the severity of transaminase elevations vary among the COVID-19 literature (29, 38, 40), and often differ from the American College of Gastroenterology (ACG) severity definitions (10). We considered these various definitions, along with known clinical information about our local population to define transaminase elevations as mild (>40 U/L and ≤ 120 U/L; >1x-3x upper limit of normal (ULN)), moderate (>120 U/L and ≤ 400 U/L; >3x-10x ULN) or severe (>400 U/L; >10x ULN).

Among composite liver scores, we included the De Ritis ratio, liver pattern based on the R index, and three non-invasive liver fibrosis scores: FIB-4, APRI, and NFS. These were calculated with the following formulas:

De Ritis ratio = $\frac{\mathrm{AST}\;\text{level}}{\mathrm{ALT}\;\text{level}}$ . Cutoffs for the De Ritis ratio were defined as: <1 (low), 1–2 (intermediate), and >2 (high) (9).

FIB-4 = $\;\frac{\mathrm{age}\times \mathrm{AST}\;\text{level}}{\text{platelet count}\times \sqrt{\mathrm{ALT}\;\text{level}}}$ . FIB-4 cutoffs were <1.3 (low), 1.3–2.67 (indeterminate), and >2.67 (high) (11, 41).

R index = $\frac{\left[\frac{\mathrm{ALT}\;\text{level}}{\mathrm{ALT}\;\text{upper limit of normal}\;(\mathrm{ULN})}\right]}{\left[\frac{\mathrm{AP}\;\text{level}}{\mathrm{AP}\;\mathrm{ULN}}\right]}$ . Low R index (<2) indicates a cholestatic pattern, intermediate R index (2–5) indicates a mixed pattern, and a high R index (>5) indicates a hepatocellular pattern (10).

$\text{APRI}=\frac{\left[(\frac{\mathrm{AST}\;\text{level}}{\mathrm{AST}\;\mathrm{ULN}}\;)\times 100\right]}{\text{platelet count}}$ . APRI cutoffs were <0.5 (low), 0.5–1 (intermediate), and >1 (high) (13, 22, 42).

$\begin{array}{l} \mathrm{NFS}=-1.675+(0.037\times \mathrm{Age})+(0.094\times \mathrm{BMI})+\\ (\begin{array}{l} 1.13\times \text{impaired fasting glucose or}\;\\ \text{diabetes}\;[\mathrm{yes}=1,\mathrm{no}=0] \end{array})+\\ {}[0.99\times (\frac{\mathrm{AST}\;\text{level}}{\mathrm{ALT}\;\text{level}})]-\\ \left(0.013\times \text{Platelet count})-(0.66\times \text{Albumin}\right) \end{array}$ (12). NFS cutoffs were <−1.455 (low), −1.455 to +0.675 (indeterminate), and > + 0.675 (high) (41).

All above formulas were calculated using the following units: age (years), BMI (kg/m^2^), and other laboratory test units listed above.

### 2.4 Subgroup analyses

**Figure 1:**
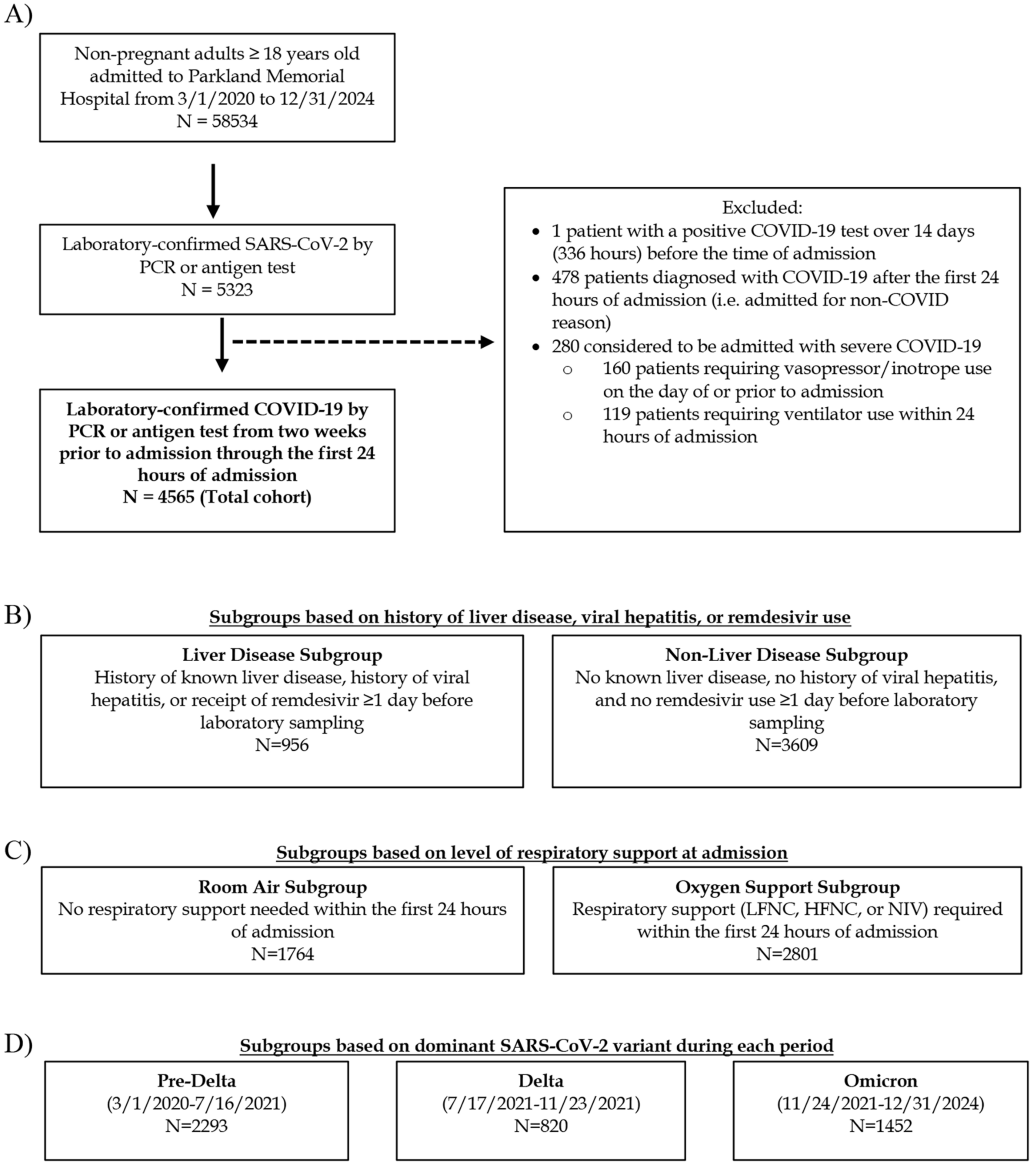
Flowchart of study cohort and subgroups. **(A)** Flow chart of patients excluded and included in the total cohort. **(B)** Separation of cohort into subgroups based on history of liver disease, viral hepatitis, or receipt of remdesivir. **(C)** Separation of cohort into subgroups based on the maximum level of respiratory support within the first 24 h of admission. **(D)** Definition of variant-predominant periods based on dates. PCR, polymerase chain reaction; LFNC, low-flow nasal cannula; HFNC, high-flow nasal cannula; NIV, non-invasive ventilation.

The study cohort was divided into various subgroups:

(1) As those with pre-existing liver disease are expected to have abnormal LFTs at baseline, we divided the patients into two subgroups: the “liver disease subgroup” and “non-liver disease subgroup” (Figure 1B). The liver disease subgroup included patients with known history of liver disease and viral hepatitis. Patients who received remdesivir at least 1 day prior to baseline lab collection were also included in this subgroup due to previous reports of remdesivir-induced transaminitis (5, 43). Patients with no prior history of liver disease, viral hepatitis, or remdesivir use were included in the non-liver disease subgroup.
(2) As oxygen saturation and respiratory support requirement at admission are known risk factors for poor COVID-19 outcomes (44–46) and COVID-19 patients with Omicron are requiring less respiratory support compared to previous variants (47–49), we also divided the study cohort into subgroups based on baseline level of respiratory support (Figure 1C). Those who remained on room air for the first 24 h of admission were in the “room air subgroup,” and those who required any type of respiratory support (from low-flow nasal cannula (LFNC) up to non-invasive ventilation (NIV)) were in the “oxygen support subgroup.”
(3) Using county- and state-level epidemiologic data (50–53), patients were divided into Pre-Delta, Delta, or Omicron subgroups according to the predominant SARS-CoV-2 variant at the time of admission: Pre-Delta (before July 17, 2021), Delta (July 17, 2021 to November 23, 2021), or Omicron (November 24, 2021 to December 31, 2024) (Figure 1D).

### 2.5 Statistical analysis

Groups were compared by Mann–Whitney-U tests or Kruskal-Wallis tests for continuous variables. Groups were compared by Pearson Chi-squared tests for categorical variables.

To identify factors associated with risk of severe COVID-19, we calculated relative risks via Poisson regression with robust error variance (54). Exposures included demographic characteristics, comorbidities, baseline laboratory values, initial respiratory support, prior remdesivir use, and composite liver scores. To determine whether elevations in LFTs or composite liver scores were independently associated with risk of severe COVID-19, multivariable regression models were adjusted for the aforementioned exposures. Collinearity was defined as a variance inflation factor (VIF) > 10 or Pearson correlation coefficient ≥0.7 or ≤ − 0.7. Collinear variables were excluded from the multivariable models. Covariates with a VIF > 10 included age, BMI, hemoglobin, and albumin. As age and obesity/BMI have been frequently associated with COVID-19 outcome in prior literature (55–58), and are basic demographic information, we included age and BMI in the multivariable models. Albumin level has frequently been reported as associated with poor COVID-19 outcome (5, 57, 59, 60) and had a stronger effect size than hemoglobin level in our preliminary analysis (data not shown); therefore, albumin was included in the multivariable models whereas hemoglobin was excluded. While BUN and CKD data were collected in this cohort, we chose to adjust for creatinine in the multivariable models for the following reasons: (1) creatinine is used to define CKD, (2) creatinine captures both acute and chronic kidney injury/dysfunction, (3) creatinine was collinear with BUN (Pearson correlation coefficient = 0.75).

All LFT elevation categories had VIFs below 10 when included in the same regression model indicating that these LFT categories were not collinear with each other (data not shown). For composite liver scores (De Ritis ratio, FIB-4, NFS, APRI, or R index (i.e., liver pattern)), individual laboratory components of those scores (AST, ALT, AP, platelets, and/or albumin depending on the score of interest) are determined by disease pathology: their production and release from tissues are due to the underlying disease which the score is designed to measure. However other patient factors such as age, BMI, and DM act as effect modifiers, and modify the magnitude of the score. Therefore, for models with composite liver score variables, individual laboratory components of the composite liver scores were not included in the models separately, whereas modifying components were adjusted for separately. For example, the models with the categorical NFS variable (i.e., high, indeterminate or low NFS) were also adjusted for age, BMI, and comorbid DM, but were not adjusted for AST elevations, ALT elevations, platelets, or albumin. Using this strategy, VIFs of the covariates and mean model VIFs were similar across the individual LFT model and the composite liver score models, and VIFs of the composite liver score variables remained below 10. See each figure legend for details regarding covariates included in each multivariable model.

Variables with over 30% missingness (LDH, direct bilirubin, PT/INR, and GGT) were excluded from all multivariable models. CRP and d-dimer also had over 30% missingness in our cohort, and only 41 and 30% of Omicron patients had available CRP and d-dimer data, respectively; therefore, CRP and d-dimer were also excluded from the main multivariable models. However, CRP and d-dimer have been reported in the literature as associated with poor COVID-19 outcomes (8, 57, 60–62). Therefore, we performed a sensitivity analysis in which the multivariable models described above were also adjusted for CRP and d-dimer; thus, the multivariable models in the sensitivity analysis (Supplementary Figure S1) only included patients with available data for CRP, d-dimer, and the other covariates described above.

The fit and discriminatory ability of the multivariable regression models were compared via the area under the receiver operating characteristic (ROC) curve and Akaike Information Criterion (AIC).

Statistical significance was set at *p* < 0.05 for all analyses. Analyses were performed using STATA SE (StataCorp LLC, College Station, TX, USA).

## 3 Results

### 3.1 Baseline characteristics

Of the 58,534 non-pregnant adult patients admitted during the study period, 4,565 were admitted with non-severe laboratory-confirmed COVID-19 diagnosed within the first 24 h of admission or earlier (Figure 1A).

Baseline characteristics of the cohort, including demographics and baseline level of respiratory support at admission are shown in Table 1. The study cohort had a median age of 55 years, was predominantly male, and the majority identified as White race and Hispanic ethnicity. The most common comorbidities were DM and hypertension. 1764 patients (38.6%) did not require oxygen support within the first 24 h of admission. Among those requiring supplemental oxygen at admission, LFNC was the modality most frequently used. Only 1.0% received remdesivir prior to laboratory testing. During the study period, 2,293 patients (50.2%), 820 patients (18.0%), and 1,452 patients (31.8%) were admitted during the Pre-Delta, Delta, and Omicron variant-predominant periods, respectively (Figure 1D and Table 1). Overall, 16.8% of patients (*N* = 766) developed severe COVID-19 and 11.5% (*N* = 525) died (Table 1). Among the surviving patients who met the definition of severe COVID-19 based on receiving organ support at some point during their admission (*N* = 241), 68.9% (*N* = 166) met the mechanical ventilation criterion, 3.7% (*N* = 9) met the ECMO criterion, 12.9% (*N* = 31) met the CRRT criterion, and 84.6% (*N* = 204) met the intravenous vasopressors or inotropes criterion. Since CRRT and ECMO start dates and times were not available, we explored which other severe COVID-19 criteria were met (if any) for patients requiring CRRT and/or ECMO. All patients who required ECMO also required mechanical ventilation and vasopressors starting at least 1 day after admission. However, 3 patients were defined as developing severe COVID-19 based solely on the CRRT criterion, as they required CRRT but did not meet any other severe COVID-19 criteria during their admission (data not shown).

**Table 1:** Study cohort baseline characteristics and COVID-19 outcome.

	Total 4,565 (100.0%)	Non-severe COVID-19 3,799 (83.2%)	Severe COVID-19766 (16.8%)	*p*-value^a^
Age	55 (43–65) (*N* = 4,565)	54 (42–64)	61 (49–70)	<0.001*
Sex Female Male	1,894 (41.5%)2,671 (58.5%)	1,607 (42.3%)2,192 (57.7%)	287 (37.5%)479 (62.5%)	0.013*
Race White Black Asian Native American/Alaska Native Other Pacific Islander Unknown	3,280 (71.9%)1,111 (24.3%)88 (1.9%)11 (0.2%)14 (0.3%)61 (1.3%)	2,673 (70.4%)983 (25.9%)70 (1.8%)8 (0.2%)13 (0.3%)52 (1.4%)	607 (79.2%)128 (16.7%)18 (2.3%)3 (0.4%)1 (0.1%)9 (1.2%)	<0.001*
Ethnicity Not Hispanic Hispanic Unknown	1,749 (38.3%)2,748 (60.2%)68 (1.5%)	1,521 (40.0%)2,215 (58.3%)63 (1.7%)	228 (29.8%)533 (69.6%)5 (0.7%)	<0.001*
BMI	29 (25–35) (*N* = 4,523)	29 (25–34)	30 (26–37)	<0.001*
Previous CV complications	1,122 (24.6%)	877 (23.1%)	245 (32.0%)	<0.001*
HTN	1,857 (40.7%)	1,541 (40.6%)	316 (41.3%)	0.723
Respiratory disease	928 (20.3%)	708 (18.6%)	220 (28.7%)	<0.001*
DM	2,201 (48.2%)	1,783 (46.9%)	418 (54.6%)	<0.001*
CKD	1,142 (25.0%)	912 (24.0%)	230 (30.0%)	<0.001*
Cancer	405 (8.9%)	310 (8.2%)	95 (12.4%)	<0.001*
Liver disease	768 (16.8%)	551 (14.5%)	217 (28.3%)	<0.001*
History of viral hepatitis	248 (5.4%)	206 (5.4%)	42 (5.5%)	0.946
HIV	165 (3.6%)	142 (3.7%)	23 (3.0%)	0.320
Rheumatologic disease	128 (2.8%)	105 (2.8%)	23 (3.0%)	0.715
Dementia	230 (5.0%)	176 (4.6%)	54 (7.0%)	0.005*
Paralysis	118 (2.6%)	93 (2.4%)	25 (3.3%)	0.194
Maximum level of respiratory support (within first 24 h of admission) Room Air LFNC HFNC NIV	1,764 (38.6%)2,117 (46.4%)523 (11.5%)161 (3.5%)	1,635 (43.0%)1,775 (46.7%)264 (6.9%)125 (3.3%)	129 (16.8%)342 (44.6%)259 (33.8%)36 (4.7%)	<0.001*
Remdesivir at least 1 day prior to lab collection	46 (1.0%)	38 (1.0%)	8 (1.0%)	0.911
Variant-predominant period Pre-Delta Delta Omicron	2,293 (50.2%)820 (18.0%)1,452 (31.8%)	1,865 (49.1%)644 (17.0%)1,290 (34.0%)	428 (55.9%)176 (23.0%)162 (21.1%)	<0.001*
COVID-19 progression				
Died	525 (11.5%)	0 (0.0%)	575 (75.1%)	N/A^b^
ICU	743 (16.3%)	168 (4.4%)	525 (68.5%)	<0.001*
LOS	7 (4–13) (*N* = 4,565)	6 (4–10)	23 (12–43)	<0.001*

a Mann-Whitney U test for continuous variables; Chi^2^ test for categorical variables.

b Part of severe COVID-19 definition.

Percentages may not add up to 100% due to rounding. Continuous variables are reported as median (interquartile range). Categorical variables are reported as N (%). An asterisk (*) indicates statistical significance (*p* < 0.05). BMI, body mass index; CV, cardiovascular; HTN, hypertension; DM, diabetes mellitus; CKD, chronic kidney disease; HIV, human immunodeficiency virus; LFNC, low-flow nasal cannula; HFNC, high-flow nasal cannula; NIV, non-invasive ventilation; ICU, intensive care unit; LOS, length of stay.

Baseline laboratory results and composite liver scores are shown in Tables 2, 3, respectively. Among patients with LFTs collected, elevations in AST, ALT, AP, and total bilirubin occurred in 52.8, 36.0, 20.4, and 10.7% of the cohort, respectively (Table 2). Less than 10% of the cohort had available direct bilirubin results and less than 1% had available GGT results. Of those who had direct bilirubin or GGT results, the majority had elevated values. FIB-4, APRI, NFS, and the De Ritis ratio were categorized as high in more than 20% of our cohort (Table 3). Per the R index, LFTs were of cholestatic pattern in more than 75% of patients. In general, elevated LFTs and composite liver scores were more frequent in severe COVID-19 compared to non-severe COVID-19 patients, respectively (Tables 2, 3).

**Table 2:** Baseline laboratory results (within 24 h of admission).

	Total 4,565 (100.0%)	Non-severe COVID-193,799 (83.2%)	Severe COVID-19766 (16.8%)	*p*-value^a^
AST (U/L)	42 (28–68) (*N* = 4,286)	40 (27–64)	55 (36–87)	<0.001*
AST Elevations Normal Mild Moderate Severe	2,024 (47.2%)1,898 (44.3%)321 (7.5%)43 (1.0%)	1,792 (50.6%)1,481 (41.8%)235 (6.6%)31 (0.9%)	232 (31.1%)417 (55.8%)86 (11.5%)12 (1.6%)	<0.001*
ALT (U/L)	30 (19–52) (*N* = 4,297)	29 (18–51)	35 (22–56)	<0.001*
ALT Elevations Normal Mild Moderate Severe	2,750 (64.0%)1,289 (30.0%)235 (5.5%)23 (0.5%)	2,315 (65.2%)1,031 (29.0%)186 (5.2%)18 (0.5%)	435 (58.2%)258 (34.5%)49 (6.6%)5 (0.7%)	0.004*
AP (U/L)	91 (71–120) (*N* = 4,296)	90 (71–119)	93 (71–129)	0.076
AP Elevation Normal Elevated	3,420 (79.6%)876 (20.4%)	2,853 (80.4%)696 (19.6%)	567 (75.9%)180 (24.1%)	0.006*
Total Bilirubin (mg/dL)	0.5 (0.3–0.7) (*N* = 4,297)	0.5 (0.3–0.7)	0.5 (0.4–0.8)	<0.001*
Total Bilirubin Elevation Normal Elevated	3,838 (89.3%)459 (10.7%)	3,184 (89.7%)366 (10.3%)	654 (87.6%)93 (12.4%)	0.085
Direct Bilirubin (mg/dL)	0.8 (0.5–1.3) (*N* = 447)	0.7 (0.4–1.2)	1.0 (0.6–1.5)	<0.001*
Direct Bilirubin Elevation Normal Elevated	34 (7.6%)413 (92.4%)	31 (8.8%)322 (91.2%)	3 (3.2%)91 (96.8%)	0.069
GGT (U/L)	138 (37–254) (*N* = 42)	157 (36–322)	65 (53–227)	0.592
GGT Elevation Normal Elevated	13 (31.0%)29 (69.0%)	10 (30.3%)23 (69.7%)	3 (33.3%)6 (66.7%)	0.862
Creatinine (mg/dL)	0.94 (0.72–1.38) (*N* = 4,556)	0.93 (0.72–1.35)	1.03 (0.77–1.57)	<0.001*
CRP (mg/dL)	9 (4–16) (*N* = 3,144)	8 (4–15)	13 (8–21)	<0.001*
D-dimer (mcg/mL)	0.85 (0.53–1.54) (*N* = 2,749)	0.84 (0.52–1.50)	0.95 (0.59–1.69)	<0.001*
Albumin (g/dL)	3.7 (3.4–4.0) (*N* = 4,299)	3.8 (3.4–4.1)	3.5 (3.2–3.8)	<0.001*
Hemoglobin (g/dL)	13.2 (11.2–14.7) (*N* = 4,552)	13.1 (11.2–14.6)	13.4 (11.3–15.0)	0.018*
Platelets (x10^9/L)	225 (170–293) (*N* = 4,552)	229 (173–301)	208 (155–268)	<0.001*
BUN (mg/dL)	16 (11–26) (*N* = 4,554)	15 (10–24)	20 (13–35)	<0.001*
LDH (U/L)	361 (262–469) (*N* = 554)	344 (255–441)	455 (336–617)	<0.001*
INR	1.2 (1.1–1.4) (*N* = 1,084)	1.2 (1.1–1.4)	1.2 (1.1–1.4)	0.014*
PT (seconds)	13.7 (12.4–15.7) (*N* = 1,084)	13.6 (12.3–15.5)	13.8 (12.8–16.6)	0.020*

a Mann-Whitney U test for continuous variables; Chi^2^ test for categorical variables.

Percentages may not add up to 100% due to rounding. Continuous variables are reported as median (interquartile range). Categorical variables are reported as N (%). An asterisk (*) indicates statistical significance (*p* < 0.05). AST, aspartate aminotransferase; ALT, alanine aminotransferase; AP, alkaline phosphatase; GGT, gamma-glutamyl transferase; CRP=C-reactive protein; BUN, blood urea nitrogen; LDH, lactate dehydrogenase; INR, international normalized ratio; PT, prothrombin time.

**Table 3:** Composite liver scores within 24 h of admission.

	Total 4,565 (100.0%)	Non-severe COVID-19 3,799 (83.2%)	Severe COVID-19766 (16.8%)	*p*-value^a^
FIB-4	1.87 (1.11–3.22) (*N* = 4,282)	1.72 (1.02–2.95)	2.71 (1.71–4.45)	<0.001*
FIB-4 risk categories Low Indeterminate High	1,323 (30.9%)1,555 (36.3%)1,404 (32.8%)	1,216 (34.4%)1,295 (36.6%)1,024 (29.0%)	107 (14.3%)260 (34.8%)380 (50.9%)	<0.001*
NFS	−0.09 (−1.58–1.35) (*N* = 4,238)	−0.28 (−1.82–1.13)	0.84 (−0.56–2.15)	<0.001*
NFS risk categories Low Indeterminate High	1,133 (26.7%)1,595 (37.6%)1,510 (35.6%)	1,038 (29.7%)1,345 (38.5%)1,112 (31.8%)	95 (12.8%)250 (33.6%)398 (53.6%)	<0.001*
APRI	0.49 (0.28–0.88) (*N* = 4,282)	0.46 (0.27–0.80)	0.70 (0.39–1.16)	<0.001*
APRI risk categories Low Intermediate High	2,174 (50.8%)1,200 (28.0%)908 (21.2%)	1,918 (54.3%)946 (26.8%)671 (19.0%)	256 (34.3%)254 (34.0%)237 (31.7%)	<0.001*
De Ritis Ratio	1.40 (1.03–1.92) (*N* = 4,286)	1.35 (1.00–1.87)	1.61 (1.20–2.18)	<0.001*
De Ritis ratio cutoff categories <1 1–2 >2	939 (21.9%)2,431 (56.7%)916 (21.4%)	834 (23.6%)2,015 (56.9%)690 (19.5%)	105 (14.1%)416 (55.7%)226 (30.3%)	<0.001*
LFT pattern Mixed Hepatocellular Cholestatic	852 (19.8%)164 (3.8%)3,279 (76.3%)	690 (19.4%)130 (3.7%)2,728 (76.9%)	162 (21.7%)34 (4.6%)551 (73.8%)	0.163

a Mann-Whitney U test for continuous variables; Chi^2^ test for categorical variables.

Percentages may not add up to 100% due to rounding. Continuous variables are reported as median (interquartile range). Categorical variables are reported as N (%). An asterisk (*) indicates statistical significance (*p* < 0.05). FIB-4, fibrosis-4 index; NFS, non-alcoholic fatty liver disease fibrosis score; APRI, AST to platelet ratio index; LFT, liver function test.

### 3.2 Association of elevated LFTs and liver scores with risk of severe COVID-19

#### 3.2.1 Total cohort: univariable regression

Univariable regression showed that age, male sex, Hispanic ethnicity, BMI, history of cardiovascular complications, pre-existing respiratory disease, DM, CKD, cancer, liver disease, dementia, higher level of baseline respiratory support, decreases in albumin, decreases in platelets, and increases in CRP, d-dimer, hemoglobin, INR, BUN, and LDH at admission were associated with increased risk of severe disease in our cohort (Supplementary Table S2). Among LFTs, AST elevations were associated with severe COVID-19 in a dose-dependent manner (RRs of 1.92, 2.34, and 2.43 for mild, moderate, and severe AST elevations, respectively). Mildly and moderately elevated ALT as well as elevated AP were also associated with severe COVID-19, although to a lesser degree (RRs < 1.4). Among liver scores, elevations in the De Ritis ratio, FIB-4, APRI, and NFS were associated with dose-dependent increases in risk of severe COVID-19, with high FIB-4 and high NFS having the greatest effect size (high FIB-4 RR: 3.35, 95% CI 2.74–4.09; high NFS RR: 3.14, 95% CI 2.55–3.88).

#### 3.2.2 Total cohort: adjusted relative risks of severe COVID-19

**Figure 2:**
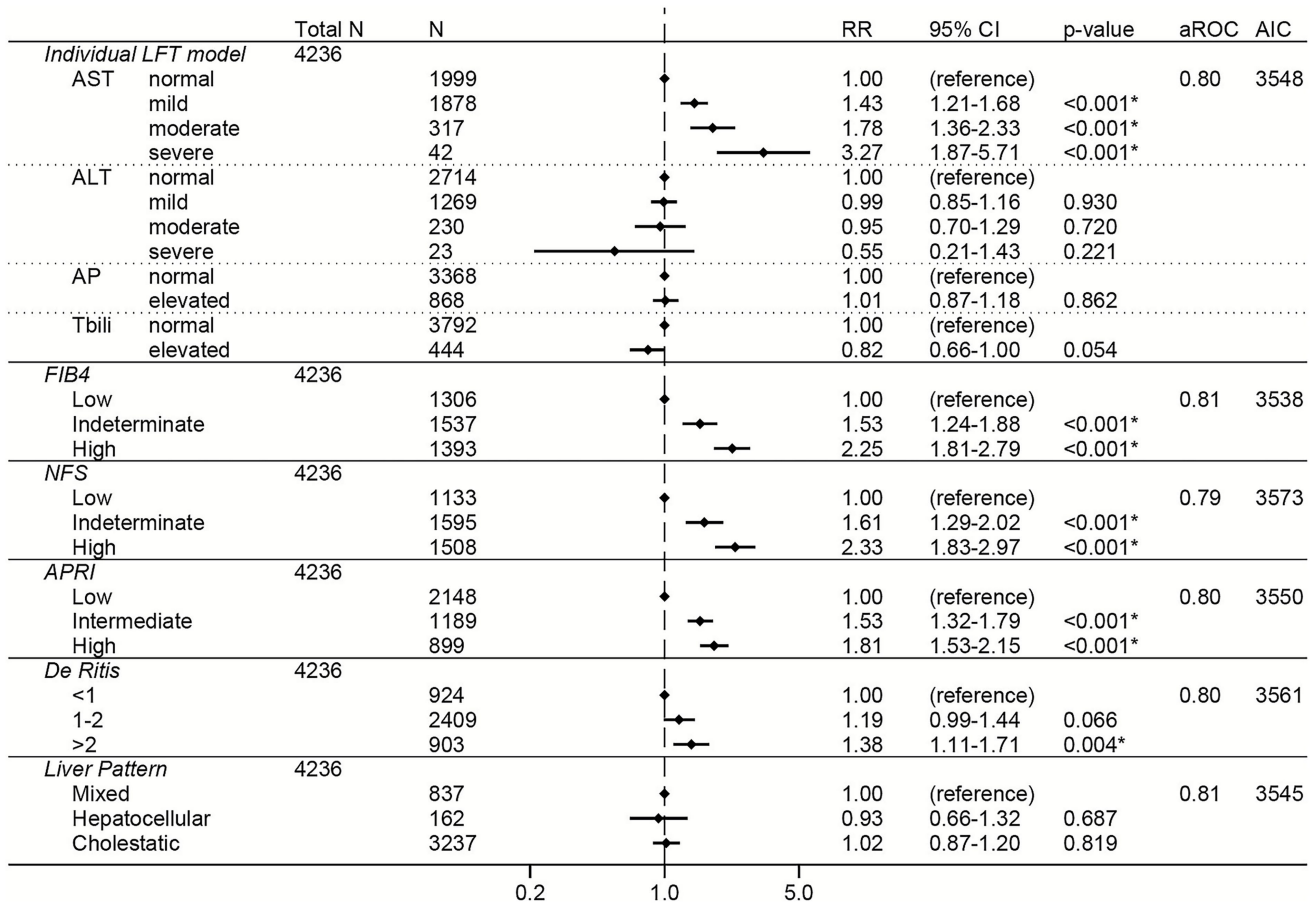
Forest plots of relative risks of severe COVID-19 in the total cohort. All models are adjusted for age, sex, race, ethnicity, body mass index, comorbidities (hypertension, cardiovascular complications, respiratory disease, diabetes mellitus, cancer, liver disease, viral hepatitis, HIV, rheumatologic disease, dementia, paralysis), baseline level of respiratory support, remdesivir prior to lab collection, and creatinine. The individual LFT model was also adjusted for albumin and platelets. The FIB-4 model was also adjusted for albumin, AP elevations, and total bilirubin elevations. The NFS model was also adjusted for AP elevations and total bilirubin elevations. The APRI model was also adjusted for ALT elevations, AP elevations, total bilirubin elevations, and albumin. The De Ritis model was also adjusted for AP elevations, total bilirubin elevations, albumin, and platelets. The liver pattern model was also adjusted for AST elevations, total bilirubin elevations, albumin, and platelets. An asterisk (*) indicates statistical significance (*p* < 0.05). AST, aspartate aminotransferase, ALT, alanine aminotransferase, AP, alkaline phosphatase, Tbili, total bilirubin; FIB4, fibrosis-4 index; NFS, non-alcoholic fatty liver disease fibrosis score; APRI, AST to platelet ratio index; RR, relative risk; aROC, area under the receiver operating characteristic curve; AIC, Akaike Information Criterion; CI, confidence interval.

Among the total cohort, elevations in AST were associated with dose-dependent increased risk of severe COVID-19 (RRs of 1.43, 1.78, and 3.27 for mild, moderate, and severe AST elevations, respectively; Figure 2). Elevations in the non-invasive liver fibrosis scores (FIB-4, NFS, and APRI) were all independently associated with dose-dependent increases in risk of severe COVID-19 (Figure 2), with high FIB4 (RR 2.25, 95% CI 1.81–2.79) and high NFS (RR 2.33, 95% CI 1.83–2.97) having the greatest effect sizes. De Ritis ratio >2 was also associated with increased risk of severe COVID-19, although with a smaller effect size (RR 1.38, 95% CI 1.11–1.71). AP elevations, ALT elevations, total bilirubin elevations, and LFT pattern were not associated with risk of severe COVID-19 (Figure 2).

The sensitivity analysis adjusting for CRP and d-dimer in addition to the other covariates had fewer observations (*N* = 2,500) but showed similar relationships between LFTs or composite liver scores and risk of severe COVID-19 (Supplementary Figure S1). High FIB-4 had the greatest effect size in this sensitivity analysis (RR 2.50, 95% CI 1.93–3.25).

#### 3.2.3 Non-liver disease subgroup

History of liver disease and viral hepatitis were present in 16.8 and 5.4% of the patients, respectively (Table 1). Elevated levels of AST, ALT, AP, total bilirubin, direct bilirubin, FIB-4, NFS, APRI, and the De Ritis ratio at admission were significantly more frequent in the liver disease subgroup compared to the non-liver disease subgroup (Supplementary Table S3). Among the composite liver scores, NFS was the most frequently elevated in the liver disease subgroup (*N* = 725, 80.2%) and the De Ritis ratio was the most frequently elevated in the non-liver disease subgroup (*N* = 2,613; 77.6%).

**Figure 3:**
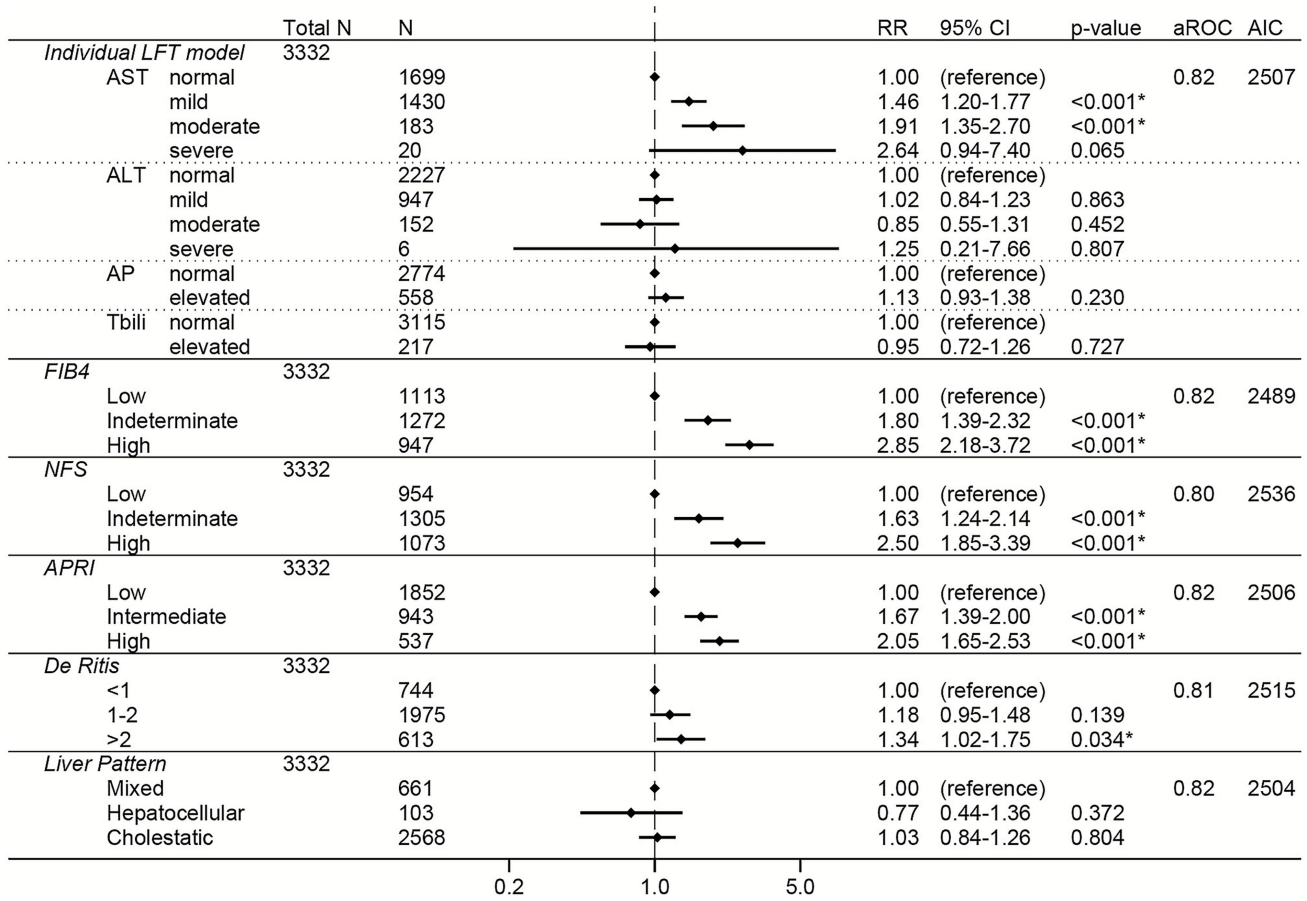
Forest plots of relative risks of severe COVID-19 in the non-liver disease subgroup (i.e., excluding patients with a history of liver disease, viral hepatitis, or receipt of remdesivir prior to lab collection). All models are adjusted for age, sex, race, ethnicity, body mass index, comorbidities (hypertension, cardiovascular complications, respiratory disease, diabetes mellitus, cancer, HIV, rheumatologic disease, dementia, paralysis), baseline level of respiratory support, and creatinine. The individual LFT model was also adjusted for albumin and platelets. The FIB-4 model was also adjusted for albumin, AP elevations, and total bilirubin elevations. The NFS model was also adjusted for AP elevations and total bilirubin elevations. The APRI model was also adjusted for ALT elevations, AP elevations, total bilirubin elevations, and albumin. The De Ritis model was also adjusted for AP elevations, total bilirubin elevations, albumin, and platelets. The liver pattern model was also adjusted for AST elevations, total bilirubin elevations, albumin, and platelets. An asterisk (*) indicates statistical significance (*p* < 0.05). AST, aspartate aminotransferase, ALT, alanine aminotransferase, AP, alkaline phosphatase, Tbili, total bilirubin; FIB4, fibrosis-4 index; NFS, non-alcoholic fatty liver disease fibrosis score; APRI, AST to platelet ratio index; RR, relative risk; aROC, area under the receiver operating characteristic curve; AIC, Akaike Information Criterion; CI, confidence interval.

Similar to the total cohort, elevations in AST, FIB-4, NFS, APRI, and the De Ritis ratio were associated with increased risk of severe COVID-19 in the non-liver disease subgroup in both the unadjusted (Supplementary Table S4) and adjusted (Figure 3) models. The greatest adjusted effect sizes were observed for high FIB-4 (RR 2.85, 95% CI 2.18–3.72) and high NFS (RR 2.50, 95% CI 1.85–3.39) (Figure 3).

#### 3.2.4 Room air subgroup

Elevations in AST and ALT were more frequent in the oxygen support subgroup (61.4 and 42.2%, respectively) compared to the room air subgroup (38.2 and 25.6%, respectively) (Supplementary Table S5). Conversely, elevations in AP and total bilirubin were more frequent in the room air subgroup (26.0 and 15.5%, respectively) compared to the oxygen support subgroup (17.0 and 7.8%, respectively) (Supplementary Table S5). FIB-4, NFS, and APRI elevations were more frequent in the oxygen support subgroup (75.0, 78.3, and 55.7%, respectively) compared to the room air subgroup (59.1, 64.6, and 38.3%, respectively).

**Figure 4:**
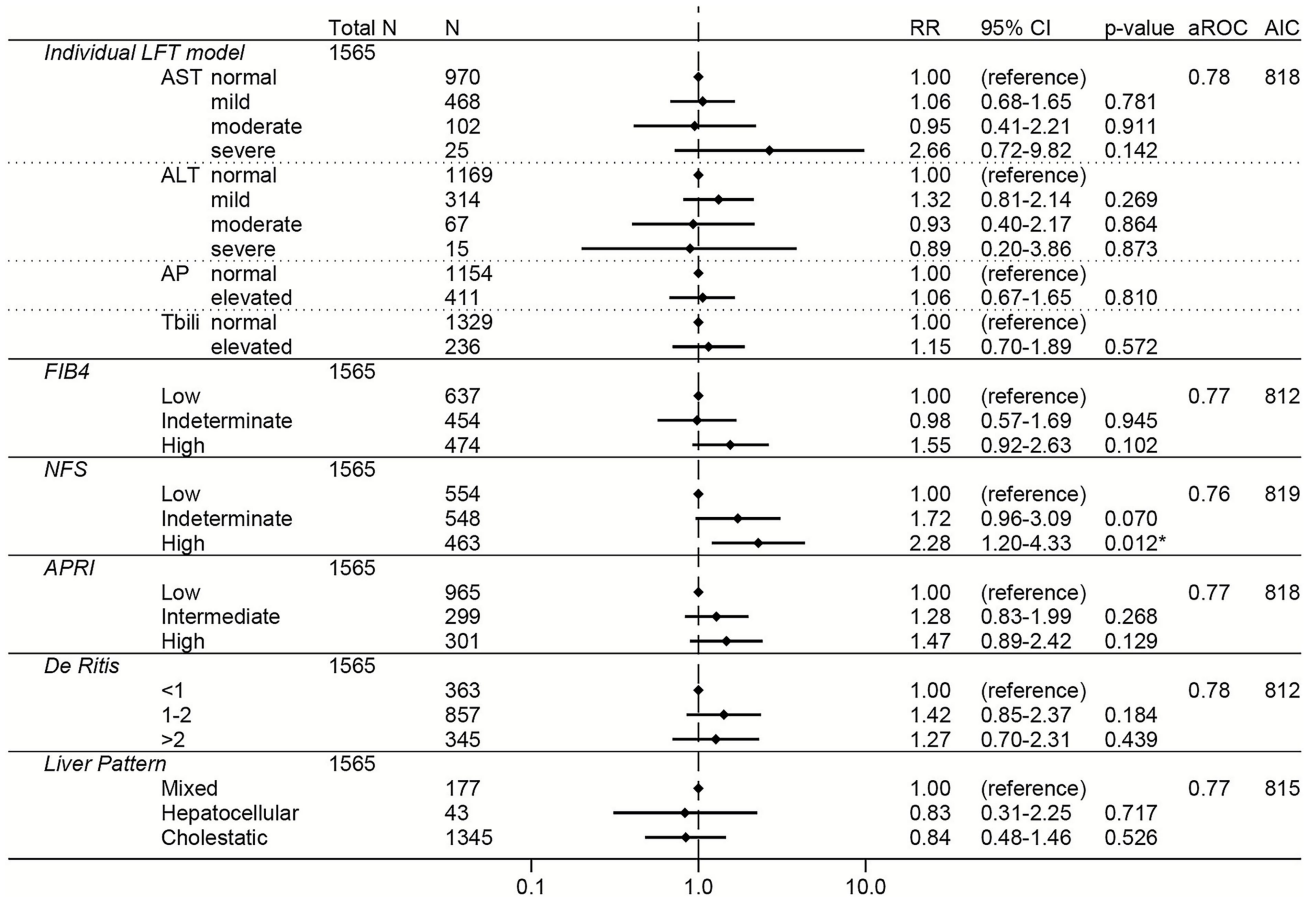
Forest plots of relative risks of severe COVID-19 in the room air subgroup (i.e., after excluding those patients who required respiratory support within the first 24 h of admission). All models are adjusted for age, sex, race, ethnicity, body mass index, comorbidities (hypertension, cardiovascular complications, respiratory disease, diabetes mellitus, cancer, liver disease, viral hepatitis, HIV, rheumatologic disease, dementia, paralysis), remdesivir prior to lab collection, and creatinine. The individual LFT model was also adjusted for albumin and platelets. The FIB-4 model was also adjusted for albumin, AP elevations, and total bilirubin elevations. The NFS model was also adjusted for AP elevations and total bilirubin elevations. The APRI model was also adjusted for ALT elevations, AP elevations, total bilirubin elevations, and albumin. The De Ritis model was also adjusted for AP elevations, total bilirubin elevations, albumin, and platelets. The liver pattern model was also adjusted for AST elevations, total bilirubin elevations, albumin, and platelets. An asterisk (*) indicates statistical significance (*p* < 0.05). AST, aspartate aminotransferase, ALT, alanine aminotransferase, AP, alkaline phosphatase, Tbili, total bilirubin; FIB4, fibrosis-4 index; NFS, non-alcoholic fatty liver disease fibrosis score; APRI, AST to platelet ratio index; RR, relative risk; aROC, area under the receiver operating characteristic curve; AIC, Akaike Information Criterion; CI, confidence interval.

The room air subgroup showed similar unadjusted results as observed in the unadjusted models for the total cohort and non-liver disease subgroups (Supplementary Table S6). However, in the adjusted models, only high NFS (RR 2.28, 95% CI 1.20–4.33) remained independently associated with increased risk of severe COVID-19 in the room air subgroup (Figure 4).

#### 3.2.5 Variant-predominant subgroups

Among the COVID-19 variant-predominant periods, AST and ALT elevations were most frequent in the Delta period (64.6 and 47.2%, respectively) whereas AP and total bilirubin elevations were most frequent in the Omicron period (25.3 and 15.5%, respectively) (Supplementary Table S7). FIB-4, the De Ritis ratio, APRI, and NFS were frequently elevated across the variant subgroups: each of these scores were elevated in at least 40% of patients in each variant subgroup. All three variants showed a predominantly cholestatic pattern of LFTs (over 65% of patients in each variant subgroup).

**Figure 5:**
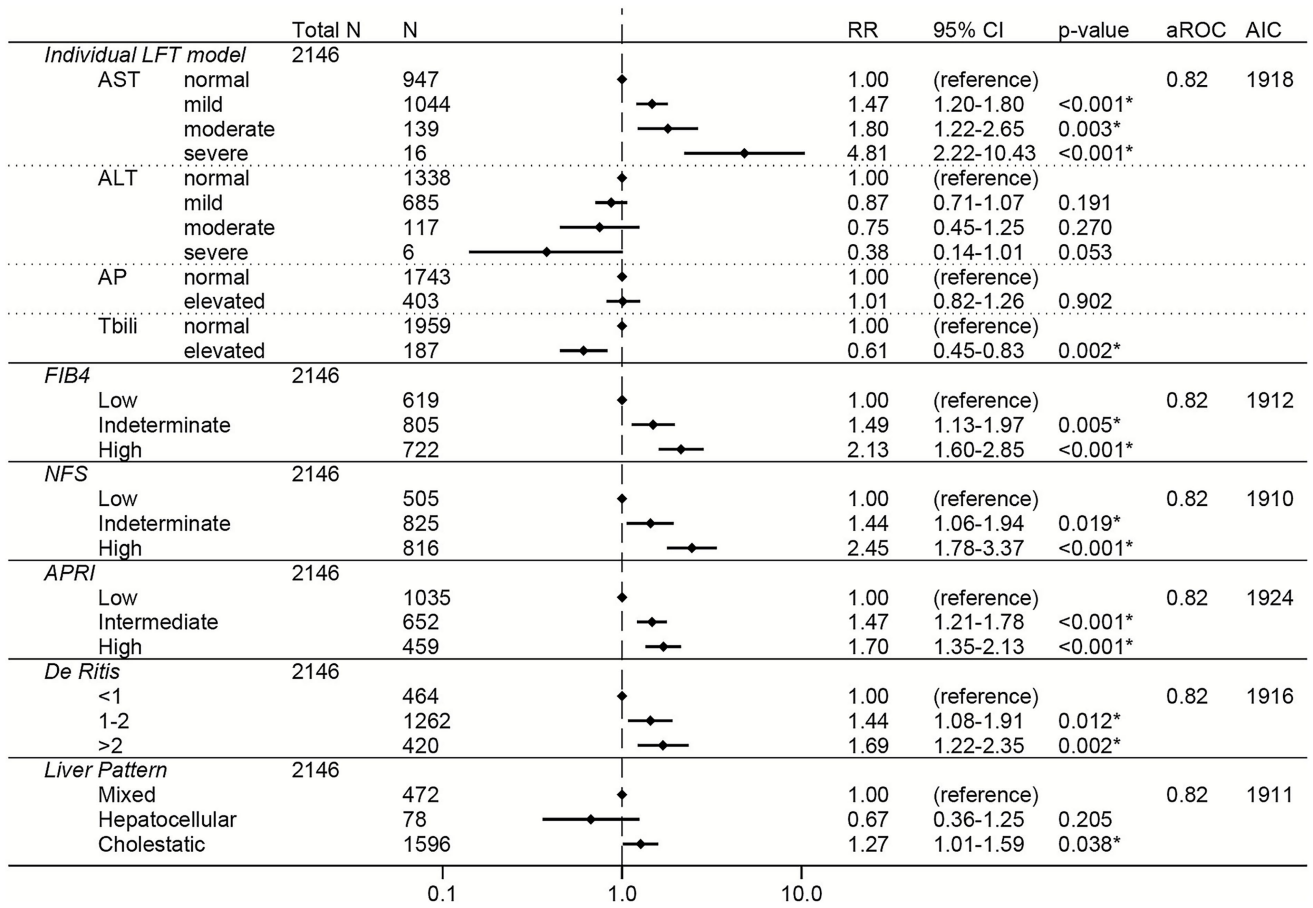
Forest plots of relative risks of severe COVID-19 during the Pre-Delta period. All models are adjusted for age, sex, race, ethnicity, body mass index, comorbidities (hypertension, cardiovascular complications, respiratory disease, diabetes mellitus, cancer, liver disease, viral hepatitis, HIV, rheumatologic disease, dementia, paralysis), baseline level of respiratory support, remdesivir prior to lab collection, and creatinine. The individual LFT model was also adjusted for albumin and platelets. The FIB-4 model was also adjusted for albumin, AP elevations, and total bilirubin elevations. The NFS model was also adjusted for AP elevations and total bilirubin elevations. The APRI model was also adjusted for ALT elevations, AP elevations, total bilirubin elevations, and albumin. The De Ritis model was also adjusted for AP elevations, total bilirubin elevations, albumin, and platelets. The liver pattern model was also adjusted for AST elevations, total bilirubin elevations, albumin, and platelets. An asterisk (*) indicates statistical significance (*p* < 0.05). AST, aspartate aminotransferase, ALT, alanine aminotransferase, AP, alkaline phosphatase, Tbili, total bilirubin; FIB4, fibrosis-4 index; NFS, non-alcoholic fatty liver disease fibrosis score; APRI, AST to platelet ratio index; RR, relative risk; aROC, area under the receiver operating characteristic curve; AIC, Akaike Information Criterion; CI, confidence interval.

**Figure 6:**
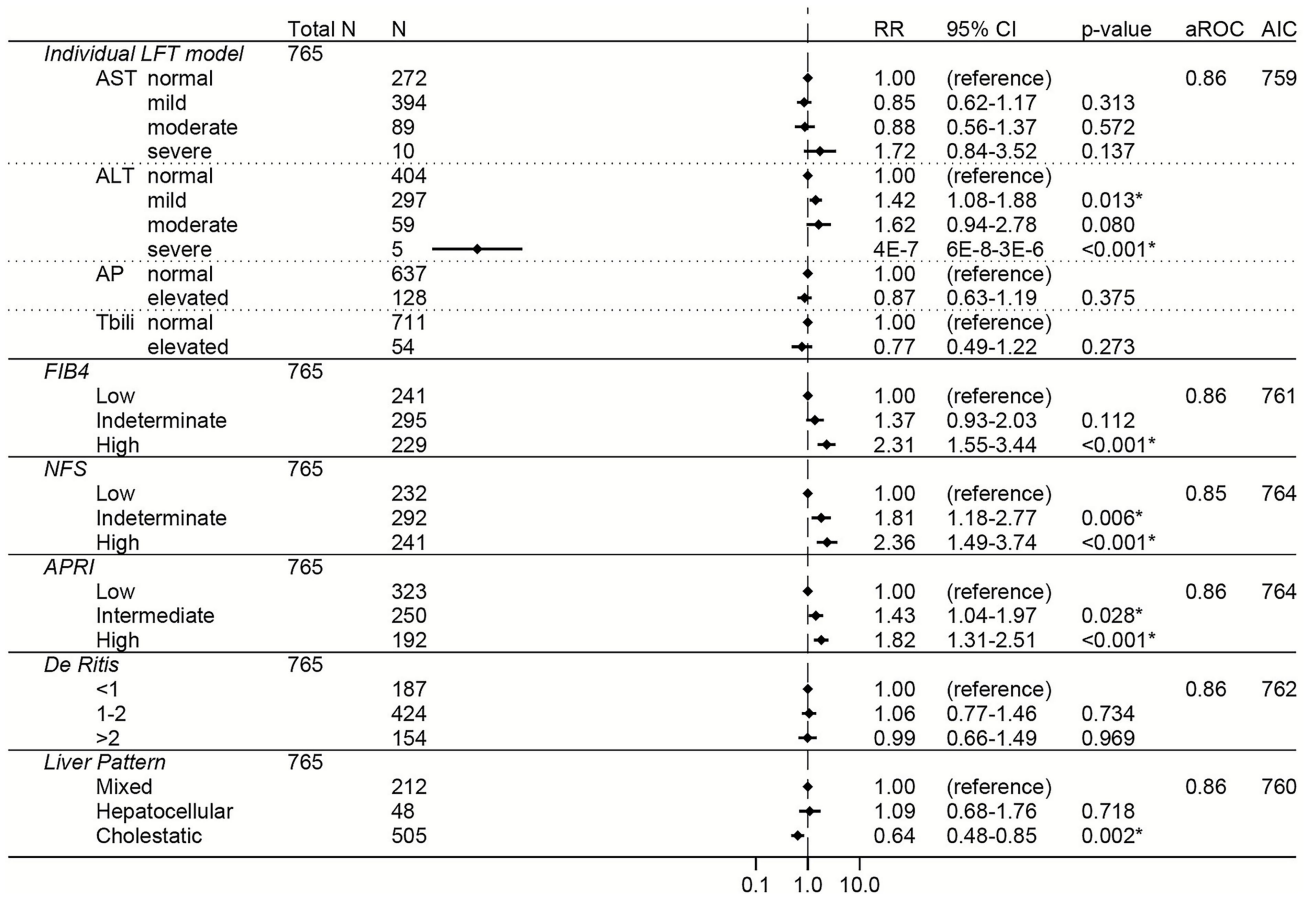
Forest plots of relative risks of severe COVID-19 during the Delta period. All models are adjusted for age, sex, race, ethnicity, body mass index, comorbidities (hypertension, cardiovascular complications, respiratory disease, diabetes mellitus, cancer, liver disease, viral hepatitis, HIV, rheumatologic disease, dementia, paralysis), baseline level of respiratory support, remdesivir prior to lab collection, and creatinine. The individual LFT model was also adjusted for albumin and platelets. The FIB-4 model was also adjusted for albumin, AP elevations, and total bilirubin elevations. The NFS model was also adjusted for AP elevations and total bilirubin elevations. The APRI model was also adjusted for ALT elevations, AP elevations, total bilirubin elevations, and albumin. The De Ritis model was also adjusted for AP elevations, total bilirubin elevations, albumin, and platelets. The liver pattern model was also adjusted for AST elevations, total bilirubin elevations, albumin, and platelets. An asterisk (*) indicates statistical significance (*p* < 0.05). AST, aspartate aminotransferase, ALT, alanine aminotransferase, AP, alkaline phosphatase, Tbili, total bilirubin; FIB4, fibrosis-4 index; NFS, non-alcoholic fatty liver disease fibrosis score; APRI, AST to platelet ratio index; RR, relative risk; aROC, area under the receiver operating characteristic curve; AIC, Akaike Information Criterion; CI, confidence interval.

**Figure 7:**
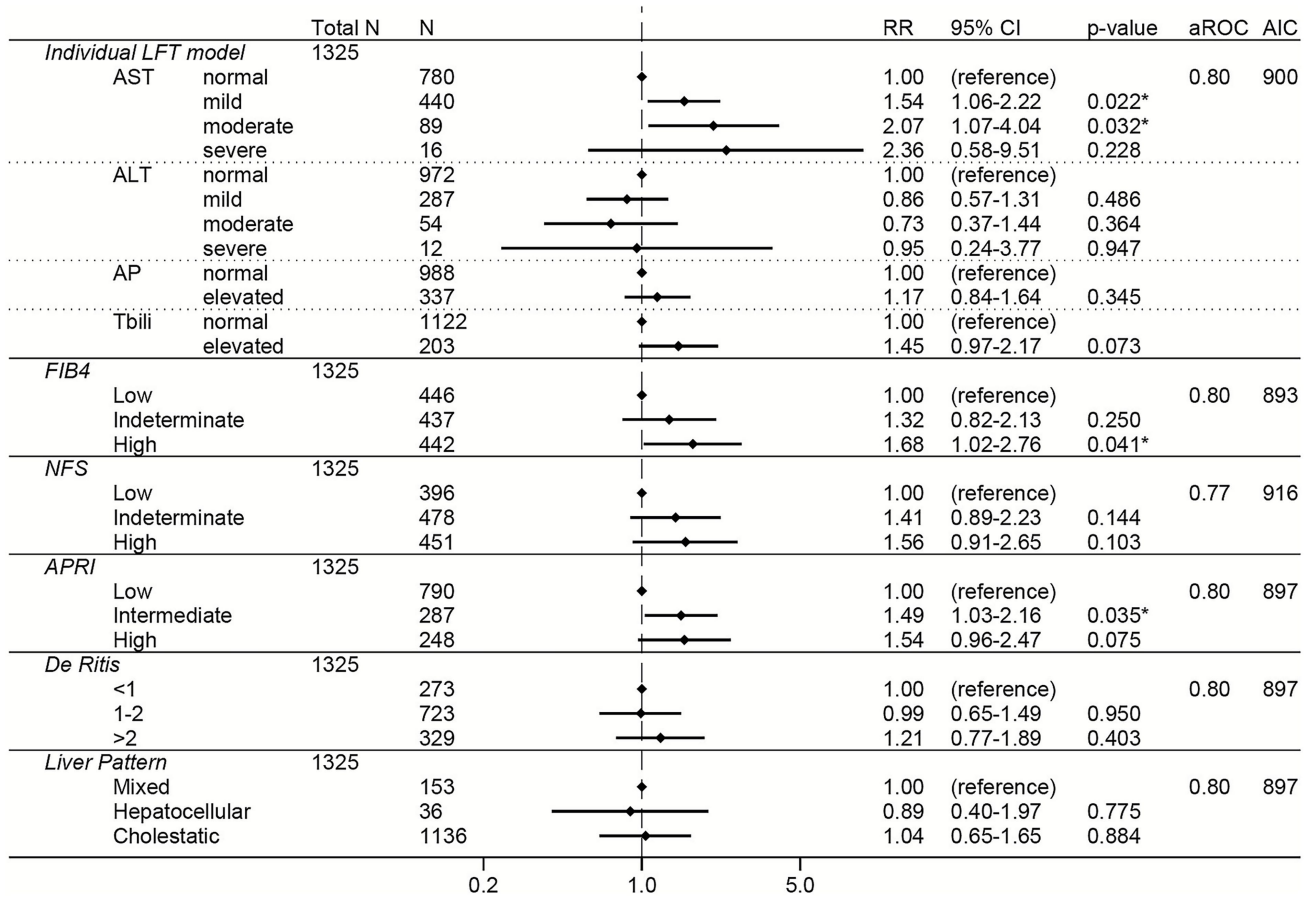
Forest plots of relative risks of severe COVID-19 during the Omicron period. All models are adjusted for age, sex, race, ethnicity, body mass index, comorbidities (hypertension, cardiovascular complications, respiratory disease, diabetes mellitus, cancer, liver disease, viral hepatitis, HIV, rheumatologic disease, dementia, paralysis), baseline level of respiratory support, remdesivir prior to lab collection, and creatinine. The individual LFT model was also adjusted for albumin and platelets. The FIB-4 model was also adjusted for albumin, AP elevations, and total bilirubin elevations. The NFS model was also adjusted for AP elevations and total bilirubin elevations. The APRI model was also adjusted for ALT elevations, AP elevations, total bilirubin elevations, and albumin. The De Ritis model was also adjusted for AP elevations, total bilirubin elevations, albumin, and platelets. The liver pattern model was also adjusted for AST elevations, total bilirubin elevations, albumin, and platelets. An asterisk (*) indicates statistical significance (*p* < 0.05). AST, aspartate aminotransferase, ALT, alanine aminotransferase, AP, alkaline phosphatase, Tbili, total bilirubin; FIB4, fibrosis-4 index; NFS, non-alcoholic fatty liver disease fibrosis score; APRI, AST to platelet ratio index; RR, relative risk; aROC, area under the receiver operating characteristic curve; AIC, Akaike Information Criterion; CI, confidence interval.

Elevations in AST, FIB-4, APRI, NFS, and the De Ritis ratio were associated with increased risk of severe COVID-19 across all three variant-predominant periods in the unadjusted models (Supplementary Table S8). The results from the adjusted models are shown for the Pre-Delta period in Figure 5, the Delta period in Figure 6, and the Omicron period in Figure 7. In the adjusted models, mild and moderate elevations in AST were associated with increased risk of severe COVID-19 during the Pre-Delta and Omicron periods (Figures 5, 7) (RRs ≥ 1.47, 95% CI lower bounds ≥1.06), but not during the Delta period (Figure 6). During the Delta period, mildly elevated ALT was associated with increased risk of severe COVID-19 (*N* = 297, RR 1.42, 95% CI 1.08–1.88) but severely elevated ALT was associated with reduced risk of severe COVID-19 (*N* = 5, RR 4E^−7^, 95% CI 6E^−8^ – 3E^−6^). Elevated total bilirubin was associated with reduced risk of severe COVID-19 during the Pre-Delta period (RR 0.61, 95% 0.45–0.83), but not during the Delta or Omicron periods.

Among the liver scores, high FIB-4 and intermediate APRI were independently associated with increased risk of severe COVID-19 across all periods, with high FIB-4 having the strongest association (high FIB-4 RRs ≥ 1.68 and 95% CI lower bounds ≥ 1.02 for all variants) (Figures 5–7).

### 3.3 Comparison of model performance

To directly compare the performance of the LFT and liver score models to each other, the area under the ROC and the AIC were calculated for each model (Figures 2–7; Supplementary Figure S1). In general, the models had similar outcome discrimination as assessed by area under ROCs, although NFS tended to have slightly lower area under ROCs compared to the other models. The FIB-4 models tended to have better (lower) AICs compared to the individual LFT, APRI, De Ritis ratio, and liver pattern models.

## 4 Discussion

Among our cohort, LFT and composite liver score elevations within 24 h of admission were common, with elevations in AST and the non-invasive liver fibrosis scores (FIB-4, NFS, and APRI) showing consistent and independent associations with severe COVID-19 in the total cohort and even in the non-liver disease subgroup (i.e., patients without pre-existing liver disease, a history of viral hepatitis, or receipt of remdesivir before baseline labs were collected). This is consistent with previous data from early studies in the pandemic (5, 14, 19–23, 25, 42, 63–71).

However, COVID-19 has changed over time with the emergence of new variants and changes in clinical practice, with poor outcomes becoming less common than early in the pandemic (26–29, 31–33, 47–49, 72, 73). Instead, it is becoming increasingly more common for COVID-19 patients to present without hypoxia and to not require oxygen at admission (28, 47, 48, 73). Ours is one of a handful of studies that investigated the association of composite liver scores with COVID-19 outcome across the different COVID-19 waves or variant-predominant periods (19, 20, 26). Furthermore, ours is one of a few studies to investigate the associations of composite liver scores with COVID-19 outcome with a focus on patients who did not require respiratory support or who did not have hypoxia at admission (15). As our cohort includes data collected as recently as December 31, 2024, our study is ideal to assess whether admission LFTs and/or composite liver scores can identify patients at increased risk of severe COVID-19 during the current COVID-19 era.

Here, we show that even in the face of these changes in COVID-19 variants and presentation over time, elevated non-invasive liver fibrosis scores at admission are associated with increased risk of severe COVID-19. It is possible that these associations may in part be due to undiagnosed liver fibrosis. However, we used a broad set of ICD-10 codes to define those with pre-existing liver disease, which included fatty liver, fibrosis, and cirrhosis, among other diagnoses (Supplementary Table S1). Even after excluding those who met a broad definition of pre-existing liver disease and/or had a history of viral hepatitis, non-invasive liver fibrosis scores remained associated with severe COVID-19 in the non-liver disease subgroup. Furthermore, the strength of the associations across all analyses, large proportion of patients in the cohort with elevated non-invasive liver fibrosis scores (over 45%), and aforementioned studies suggest that non-invasive liver fibrosis scores (FIB-4, NFS, and APRI) are independently associated with increased risk of severe COVID-19 regardless of pre-existing liver fibrosis (14, 19–23, 25, 26, 42, 63–71).

The FIB-4 models generally had the best combination of discriminatory power, effect size, and fit; however, the differences between the effect size and discriminatory power of the NFS and APRI models compared to the FIB-4 models were slight. This suggests that the differences between the FIB-4, NFS, and APRI models compared to each other are mainly statistical due to the slightly better fit of the FIB-4 models to the data analyzed here. Therefore, non-invasive liver fibrosis scores measured at admission may be useful tools for clinicians in the current COVID-19 era to identify COVID-19 patients at high risk of progressing to severe disease who may benefit from a higher level of care early during their admission.

As our data show, liver dysfunction in COVID-19 continues to be associated with risk of COVID-19 severity even as the virus evolves. However, the underlying mechanism of COVID-19-associated liver injury is complex and still not fully understood. It has been suggested that SARS-CoV-2 can directly infect liver progenitor cells, cholangiocytes, and hepatocytes, partially mediated by the presence of angiotensin-converting enzyme 2 expression, which may lead to cholestasis and hepatic dysfunction (5, 74). Indirect mechanisms of COVID-19-associated liver dysfunction are hypothesized to include cytokine storm, endothelial dysfunction/hypercoagulable state, ischemia/hypoxia, and drug-induced liver injury (5, 74).

Elevated ALT is widely recognized as a specific marker for liver damage due to its high activity in the liver relative to other organs (10, 75). Many studies have shown an association of ALT elevations with poor COVID-19 outcome (5). In our study, ALT elevations had variable associations with severe COVID-19, particularly during the Delta period. A handful of studies have shown lower ALT levels to be associated with poor COVID-19 outcome (76–78), and low ALT is suggested to be a marker of frailty and sarcopenia in non-COVID settings (79–81). Perhaps, the variability in association between ALT levels and COVID-19 outcome is due to levels of ALT being indicative of overall reserve and liver dysfunction. AST is less specific for liver dysfunction than ALT because AST is produced in other tissues such as the heart and muscles in addition to the liver (10, 75). Thus, increased AST can be a marker of direct liver injury as well as a marker of other organ dysfunction (5, 10, 74, 75). Among baseline LFTs, AST had the greatest and most consistent effect on risk of poor COVID-19 outcome, with AST elevations being consistently associated with increased risk of severe COVID-19 in our cohort and across the subgroups, consistent with many previous studies (5, 8, 78). Surprisingly, elevated total bilirubin was associated with reduced risk of severe COVID-19 during the Pre-Delta period. While non-specific, bilirubin is frequently elevated in cholestasis (10, 75). Bilirubin has antioxidant properties, with some evidence of association of increases in bilirubin with reduced mortality in chronic obstructive pulmonary disease (COPD), reduced development of cardiovascular and metabolic diseases, and other potential benefits (82, 83). Bilirubin may have a similar effect in COVID-19, as bilirubin elevations were actually more frequent in the room air subgroup compared to the oxygen support subgroup. Taken together, these results may indicate that in our cohort, abnormal LFTs were predominantly a result of indirect liver injury and/or systemic dysfunction. However, investigation of these mechanisms was beyond the scope of this study.

In this single-center study conducted at a safety-net hospital, non-liver-related variables including age, sex, BMI, various comorbidities, respiratory support requirement at admission, increases in creatinine, decreases in platelets, and decreases in albumin were associated with severe COVID-19 in our cohort, consistent with previous studies (8, 27, 33, 44–46, 55–59, 61). Furthermore, the liver-related results were largely consistent across different variant periods and baseline patient presentations in the subgroup analyses. However, given that baseline LFTs as well as the dominant variant or sub-variant can vary across populations, validation studies in other current COVID-19 cohorts in different settings are warranted.

Our study has several limitations. We did not correct for multiple comparisons, which could have led to spurious statistically significant results. Reassuringly, the results of this study are consistent with previous literature as described above. The retrospective design relied on information documented in medical records which may contain errors or incomplete data; thus, misclassifications of exposures and/or outcome are possible. For instance, we lacked data on home medications, vaccination status, and pre-COVID LFTs, making it challenging to determine if the reported abnormalities were solely due to acute COVID-19. Similarly, ECMO and CRRT start times were not available. Therefore, it is possible that the 3 patients who received CRRT but did not meet any other severe COVID-19 criteria may have received CRRT within 24 h of admission and mistakenly been included in the cohort. Since we excluded patients who received vasopressors or required mechanical ventilation within the first day of admission, and all patients requiring ECMO also received vasopressors or were mechanically ventilated at some point, it is unlikely that initiation of ECMO was a source of misclassification bias. Additionally, just as SARS-CoV-2 evolved over time during the study period (2020–2024), there were also changes in standard treatment and vaccination availability and uptake that could have potentially confounded our results. However, the consistency of the results across the variant-predominant subgroups and the room air subgroup are reassuring against this possible source of confounding. Our study was not designed to investigate the mechanism of liver pathology during COVID-19; additional prospective and/or basic science studies are necessary to clarify the mechanism(s). We did not have viral sequence data available to directly identify the circulating viral variants in our cohort, although previous studies have used similar dating methods to categorize variants (33, 72, 73). Future prospective studies should be performed to validate the prognostic role of non-invasive liver fibrosis scores across SARS-CoV-2 variants and baseline presentations of COVID-19 patients among other populations during the current Omicron period.

Regardless of the limitations, our study has several strengths. The study period was one of the longest to our knowledge in the COVID-19 literature (from March 2020 through the end of 2024) with a relatively high N for a single center study. This allowed for a robust and comprehensive analysis of abnormal LFTs and composite liver scores during the COVID-19 era as a whole. This also allowed us to compare and contrast these associations during different variant-predominant periods and across different types of patients (i.e., those who present without requiring respiratory support, those without pre-existing liver disease, etc.). We also assessed model discriminatory ability and data fit via area under the ROCs and AICs, respectively to aid in the comparison of the various models presented here.

## 5 Conclusion

COVID-19 continues to present as a systemic disease with extrapulmonary signs and symptoms. Measuring LFTs and calculating non-invasive liver fibrosis scores at admission, particularly FIB-4, can help identify COVID-19 patients who do not present “classically,” but are nonetheless at increased risk of severe disease, and may benefit from a higher level of care and monitoring early in their admission.

## Data Availability

The datasets presented in this article are not readily available because this is patient-level data with PHI which cannot be made publicly available. Requests for a limited de-identified dataset can be made to the corresponding author (Nicole Naiman, nicole.naiman@utsouthwestern.edu).
