# Supplementary material for: Elevated non-invasive liver fibrosis scores at admission are independent risk factors for severe COVID-19: a retrospective cohort study from 2020 to 2024
Source: Front Med (Lausanne). 2026 Jan 5;12:1727318. doi: 10.3389/fmed.2025.1727318 (PMC12812602; doi:10.3389/fmed.2025.1727318)
Supplement: Supplementary file 1 [file Data_Sheet_1.docx]

STROBE Statement for cohort studies

|  | Item No. | Recommendation | Page  No. | Relevant text from manuscript |
| --- | --- | --- | --- | --- |
| **Title and abstract** | 1 | (*a*) Indicate the study’s design with a commonly used term in the title or the abstract | p.1 | “a retrospective cohort study from 2020 to 2024” |
|  |  |  | p.1 | “This was a retrospective cohort study” |
|  |  | (*b*) Provide in the abstract an informative and balanced summary of what was done and what was found | p.1 | “Methods: This was a …had the best fit based on AICs.” |
| Introduction | | | |  |
| Background/rationale | 2 | Explain the scientific background and rationale for the investigation being reported | p.2 | “Coronavirus disease 2019 (COVID-19) emerged…hospitalization and death still persist.” |
| Objectives | 3 | State specific objectives, including any prespecified hypotheses | p.2 | “In this retrospective cohort study, we examined the association … with different variants and disease presentations. |
| Methods | | | |  |
| Study design | 4 | Present key elements of study design early in the paper | p.2 | “This retrospective cohort study was performed with electronic medical record data from … first COVID-19 admission available per patient.” |
| Setting | 5 | Describe the setting, locations, and relevant dates, including periods of recruitment, exposure, follow-up, and data collection | p.2 | “This retrospective cohort study was performed with electronic medical record data from Parkland Memorial Hospital in Dallas, Texas from March 1, 2020, to December 31, 2024.” |
|  |  |  | p.2 | “The primary outcome was severe… ECMO and CRRT start dates and times were not available.” |
|  |  |  | p.2-3 | “Demographic information comprising age, sex, race… blood urea nitrogen (BUN; mg/dL) results were analyzed.” |
|  |  |  | p.4 | “Exposures included demographic characteristics, comorbidities, baseline laboratory values, initial respiratory support, prior remdesivir use, and composite liver scores.” |
| Participants | 6 | (*a*) *Cohort study*—Give the eligibility criteria, and the sources and methods of selection of participants. Describe methods of follow-up | p.2 | “This retrospective cohort study was performed with electronic medical record data …. We analyzed the first COVID-19 admission available per patient.” |
|  |  | (*b*) *Cohort study*—For matched studies, give matching criteria and number of exposed and unexposed | N/A |  |
| Variables | 7 | Clearly define all outcomes, exposures, predictors, potential confounders, and effect modifiers. Give diagnostic criteria, if applicable | p.2 | “The primary outcome was severe COVID-19 defined by end-organ failure (indicated by requirement of organ support) and/or mortality associated with the COVID-19 admission. For the purposes of this study, end-organ failure was defined as the use of mechanical ventilation, intravenous vasopressors or inotropes, extracorporeal membrane oxygenation (ECMO) or continuous renal replacement therapy (CRRT; not including routine dialysis) during the COVID-19 admission. Mortality associated with the COVID-19 admission was defined by a date of death in the electronic medical record from any time during the admission through 7 days after the discharge date.” |
|  |  |  | p.2-3 | “Demographic information comprising age, sex, race…and other laboratory test units listed above” |
|  |  |  | p.5 | “However other patient factors such as age, BMI, and DM act as effect modifiers, and modify the magnitude of the score.” |
| Data sources/ measurement | 8* | For each variable of interest, give sources of data and details of methods of assessment (measurement). Describe comparability of assessment methods if there is more than one group | p.2 | “electronic medical record data” |
|  |  |  | p.2-3 | “ICD-10 codes, including…NFS cutoffs were -1.455 (low), -1.455 to +0.675 (indeterminate), and >+0.675 (high)” |
| Bias | 9 | Describe any efforts to address potential sources of bias | p.3 | BMI <10 (kg/m^2^) or >100 (kg/m^2^) were assumed to be improperly entered into the medical record and considered missing (N=42).” |
|  |  |  | p.4-5 | “To determine whether elevations in LFTs or composite liver scores were independently associated … d-dimer, and the other covariates described above.” |
|  |  |  | p.13-14 | Acknowledge limitations to the study: “We did not correct for multiple comparisons, … dating methods to categorize variants” |
| Study size | 10 | Explain how the study size was arrived at | p.2 | We took all-comers based on the following criteria: “This retrospective cohort study was performed with electronic medical record data from Parkland Memorial Hospital in Dallas, Texas from March 1, 2020, to December 31, 2024. Adults ≥ 18 years old admitted with COVID-19 were included in this study. COVID-19 was defined as a positive SARS-CoV-2 reverse transcriptase polymerase chain reaction (PCR) or antigen test within 2 weeks prior to and through the first 24 h of admission. Pregnant women, outpatients, and those discharged from the emergency department were not included in this study. We analyzed the first COVID-19 admission available per patient.” |
| Quantitative variables | 11 | Explain how quantitative variables were handled in the analyses. If applicable, describe which groupings were chosen and why | p.3 | “BMI <10 (kg/m^2^) or >100 (kg/m^2^) were assumed to be improperly entered into the medical record and considered missing (N=42).” |
|  |  |  | p.3 | “Some patients had multiple results… other laboratory test units listed above.” |
|  |  |  | p.4 | “Groups were compared by Mann-Whitney-U tests or Kruskal-Wallis tests for continuous variables.” |
|  |  |  | p.4-5 | “Collinearity was defined as a variance … and the other covariates described above.” |
|  |  |  |  |  |
|  |  |  |  |  |
| Statistical methods | 12 | (*a*) Describe all statistical methods, including those used to control for confounding | p.4-5 | “Groups were compared by Mann-Whitney-U tests …STATA SE” |
|  |  | (*b*) Describe any methods used to examine subgroups and interactions | p.3 | “The study cohort was divided into various subgroups…(Figure 1D).” |
|  |  | (*c*) Explain how missing data were addressed | p.5 | “Variables with over 30% missingness… and the other covariates described above.” |
|  |  | (*d*) *Cohort study*—If applicable, explain how loss to follow-up was addressed | N/A |  |
|  |  | (*e*) Describe any sensitivity analyses | p.5 | “we performed a sensitivity analysis … other covariates described above.” |
| Results | | | | |
| Participants | 13* | (a) Report numbers of individuals at each stage of study—eg numbers potentially eligible, examined for eligibility, confirmed eligible, included in the study, completing follow-up, and analysed | p.5 | “Of the 58,534 non-pregnant adults… earlier (Figure 1A)” |
|  |  |  | Figure 1 | Figure 1 |
|  |  | (b) Give reasons for non-participation at each stage | p.5 | “Of the 58,534 non-pregnant adults… earlier (Figure 1A)” |
|  |  |  | Figure 1 | Figure 1 |
|  |  | (c) Consider use of a flow diagram | Figure 1 | Figure 1 |
| Descriptive data | 14* | (a) Give characteristics of study participants (eg demographic, clinical, social) and information on exposures and potential confounders | p.5 | “Baseline characteristics of the cohort… non-severe COVID-19 patients, respectively (Tables 2, 3).” |
|  |  |  | Tables 1-3 | Tables 1-3 |
|  |  | (b) Indicate number of participants with missing data for each variable of interest | Tables 1-3 | Tables 1-3, see Ns in the “total” column for patients with available data |
|  |  | (c) *Cohort study*—Summarise follow-up time (eg, average and total amount) | p.2 | “The primary outcome was severe COVID-19 defined by end-organ failure (indicated by requirement of organ support) and/or mortality associated with the COVID-19 admission. For the purposes of this study, end-organ failure was defined as the use of mechanical ventilation, intravenous vasopressors or inotropes, extracorporeal membrane oxygenation (ECMO) or continuous renal replacement therapy (CRRT; not including routine dialysis) during the COVID-19 admission. Mortality associated with the COVID-19 admission was defined by a date of death in the electronic medical record from any time during the admission through 7 days after the discharge date.” |
|  |  |  | Table 1 | LOS (length of stay) row |
| Outcome data | 15* | *Cohort study*—Report numbers of outcome events or summary measures over time | p. 5 | *“*Overall, 16.8% of patients (N=766) developed severe COVID-19… during their admission (data not shown).” |
|  |  |  | Table 1 | “Severe COVID-19” column; “Died” row |
| Main results | 16 | (*a*) Give unadjusted estimates and, if applicable, confounder-adjusted estimates and their precision (eg, 95% confidence interval). Make clear which confounders were adjusted for and why they were included | p.5, 7 | “**3.2.1 Total cohort: univariable regression**  Univariable regression showed that age… high NFS RR: 3.14, 95% CI 2.55-3.88).” |
|  |  |  | Supplementary Table S2 | Supplementary Table S2 |
|  |  |  | p.8 | “**3.2.2 Total cohort: adjusted relative risks of severe COVID-19…** not associated with risk of severe COVID-19 (Figure 2).” |
|  |  |  | Figure 2 | Figure 2 |
|  |  | (*b*) Report category boundaries when continuous variables were categorized | p.3 | “Definitions of normal…severe (>400 U/L; >10x ULN).” |
|  |  |  | p.3 | “Cutoffs for the De Ritis ratio were defined as: <1 (low), 1-2 (intermediate), and >2 (high)” |
|  |  |  | p.3 | “FIB-4 cutoffs were <1.3 (low), 1.3-2.67 (indeterminate), and >2.67 (high)” |
|  |  |  | p.3 | “Low R index (<2) indicates a cholestatic pattern, intermediate R index (2-5) indicates a mixed pattern, and a high R index (>5) indicates a hepatocellular pattern” |
|  |  |  | p.3 | “APRI cutoffs were <0.5 (low), 0.5-1 (intermediate), and >1 (high)” |
|  |  |  | p.3 | “NFS cutoffs were <-1.455 (low), -1.455 to +0.675 (indeterminate), and >+0.675 (high)” |
|  |  | (*c*) If relevant, consider translating estimates of relative risk into absolute risk for a meaningful time period | N/A |  |
| Other analyses | 17 | Report other analyses done—eg analyses of subgroups and interactions, and sensitivity analyses | p.8-10 | “**3.2.3 Non-liver disease subgroup**…liver pattern models.” |
| Discussion | | | | |
| Key results | 18 | Summarise key results with reference to study objectives | p.10 | “Among our cohort, LFT and composite liver score elevations within 24 h of admission were common, with elevations in AST and the non-invasive liver fibrosis scores (FIB-4, NFS, and APRI) showing consistent and independent associations with severe COVID-19 in the total cohort and even in the non-liver disease subgroup (i.e., patients without pre-existing liver disease, a history of viral hepatitis, or receipt of remdesivir before baseline labs were collected).” |
|  |  |  | p.10-11 | “Ours is one of…current COVID-19 era” |
|  |  |  | p.11 | “The FIB-4 models generally had the best combination of discriminatory power, effect size, and fit; however, the differences between the effect size and discriminatory power of the NFS and APRI models compared to the FIB-4 models were slight. This suggests that the differences between the FIB-4, NFS, and APRI models compared to each other are mainly statistical due to the slightly better fit of the FIB-4 models to the data analyzed here. Therefore, non-invasive liver fibrosis scores measured at admission may be useful tools for clinicians in the current COVID-19 era to identify COVID-19 patients at high risk of progressing to severe disease who may benefit from a higher level of care early during their admission.” |
|  |  |  | p.12 | “As our data show, liver dysfunction in COVID-19 continues to be associated with risk of COVID-19 severity even as the virus evolves.” |
| Limitations | 19 | Discuss limitations of the study, taking into account sources of potential bias or imprecision. Discuss both direction and magnitude of any potential bias | p.13-14 | “Our study has several limitations…categorize variants.” |
| Interpretation | 20 | Give a cautious overall interpretation of results considering objectives, limitations, multiplicity of analyses, results from similar studies, and other relevant evidence | p.14 | “Measuring LFTs…monitoring early in their admission.” |
| Generalisability | 21 | Discuss the generalisability (external validity) of the study results | p.13 | “In this single-center study…are warranted.” |
| Other information |  | | | |
| Funding | 22 | Give the source of funding and the role of the funders for the present study and, if applicable, for the original study on which the present article is based | p.15 | “This work was supported with funds from…design or analysis.” |

*Give information separately for cases and controls in case-control studies and, if applicable, for exposed and unexposed groups in cohort and cross-sectional studies.: **N/A**

**Note:** An Explanation and Elaboration article discusses each checklist item and gives methodological background and published examples of transparent reporting. The STROBE checklist is best used in conjunction with this article (freely available on the Web sites of PLoS Medicine at http://www.plosmedicine.org/, Annals of Internal Medicine at http://www.annals.org/, and Epidemiology at http://www.epidem.com/). Information on the STROBE Initiative is available at www.strobe-statement.org.

Copyright: © 2007 von Elm et al. Checklist utilized here based on the open-access creative commons attribution license permitting unrestricted use and reproduction.

Source of STROBE checklist:

von Elm E, Altman DG, Egger M, Pocock SJ, Gotzsche PC, Vandenbroucke JP, et al. The Strengthening the Reporting of Observational Studies in Epidemiology (STROBE) statement: guidelines for reporting observational studies. PLoS Med. 2007;4(10):e296.
