# Supplementary material for: Elevated non-invasive liver fibrosis scores at admission are independent risk factors for severe COVID-19: a retrospective cohort study from 2020 to 2024
Source: Front Med (Lausanne). 2026 Jan 5;12:1727318. doi: 10.3389/fmed.2025.1727318 (PMC12812602; doi:10.3389/fmed.2025.1727318)
Supplement: Supplementary file 2 [file Supplementary_file_1.docx]

Supplementary Material

# Supplementary Tables

## Table S1. Reference list of ICD-10 codes for comorbidities

| **Comorbidity** | **ICD-10** |
| --- | --- |
| Viral hepatitis | B15-B19, O98.4 |
| Human immunodeficiency virus (HIV) | B20-B24, Z21, F02.4, O98.7 |
| Hypertension | I10, I15, I1A |
| Prior cardiovascular complications | Patients with at least one of the following: |
| Hypertensive heart disease | I11 |
| Myocardial infarction | I21-I23 |
| Heart failure | I50 |
| Peripheral arterial disease | I73.9 |
| Cerebral infarction/ischemic stroke | I63 |
| Chronic or non-specified respiratory disease | Patients with at least one of the following: |
| Chronic or non-specified bronchitis | J40-42 |
| Emphysema | J43, J98.2, J98.3 |
| Chronic obstructive pulmonary disease (COPD) | J44 |
| Asthma | J45-J46, J82.83 |
| Bronchiectasis | J47 |
| Lung diseases due to external agents | J60-70 |
| Chronic pulmonary edema | J81.1 |
| Chronic eosinophilic pneumonia | J82.81 |
| Other pulmonary eosinophilia, not classified | J82.89 |
| Alveolar and parieto-alveolar conditions | J84.0 |
| Interstitial pulmonary/lung diseases | J84.1, J84.8, J84.9 |
| Chronic respiratory failure | J96.1 |
| Malignancy | C00-96 |
| Liver disease (includes hepatic transplant, ascites, esophageal varices) | K70-77, Z94.4, T86.4, I85, R18 |
| Diabetes mellitus | E08-E13 |
| Chronic kidney disease | N18, I12-I13, D63.1, E08.22, E09.22, E10.22, E11.22, E13.22 |
| Rheumatologic diseases | M04-M06, M08, M30-M36, M45 |
| Dementia | F01-F03, G30 |
| Paralytic syndromes | G80.0, G80.1, G80.2, G81-G83 |

## Table S2. Unadjusted relative risk of severe COVID-19 in the total cohort

|  | **N** | **RR** | **95% CI** | **p-value** |
| --- | --- | --- | --- | --- |
| Age | 4,565 | 1.02 | 1.02-1.02 | <0.001* |
| Sex | 4,565 |  |  |  |
| Female | 1,894 | 1.00 | (reference) | . |
| Male | 2,671 | 1.18 | 1.04-1.35 | 0.014* |
| Race | 4,565 |  |  |  |
| White | 3,280 | 1.00 | (reference) |  |
| Black | 1,111 | 0.62 | 0.52-0.74 | <0.001* |
| Asian | 88 | 1.11 | 0.73-1.68 | 0.639 |
| Native American/Alaska Native | 11 | 1.47 | 0.56-3.88 | 0.432 |
| Other Pacific Islander | 14 | 0.39 | 0.06-2.56 | 0.324 |
| Unknown | 61 | 0.80 | 0.43-1.46 | 0.465 |
| Ethnicity | 4,565 |  |  |  |
| Not Hispanic | 1,749 | 1.00 | (reference) |  |
| Hispanic | 2,748 | 1.49 | 1.29-1.72 | <0.001* |
| Unknown | 68 | 0.56 | 0.24-1.32 | 0.188 |
| BMI | 4,523 | 1.01 | 1.01-1.02 | <0.001* |
| Previous CV Complications | 4,565 |  |  |  |
| No | 3,443 | 1.00 | (reference) |  |
| Yes | 1,122 | 1.44 | 1.26-1.65 | <0.001* |
| HTN | 4,565 |  |  |  |
| No | 2,708 | 1.00 | (reference) |  |
| Yes | 1,857 | 1.02 | 0.90-1.17 | 0.723 |
| History of Respiratory Disease | 4,565 |  |  |  |
| No | 3,637 | 1.00 | (reference) |  |
| Yes | 928 | 1.58 | 1.37-1.81 | <0.001* |
| DM | 4,565 |  |  |  |
| No | 2,364 | 1.00 | (reference) |  |
| Yes | 2,201 | 1.29 | 1.13-1.47 | <0.001* |
| CKD | 4,565 |  |  |  |
| No | 3,423 | 1.00 | (reference) |  |
| Yes | 1,142 | 1.29 | 1.12-1.48 | <0.001* |
| Cancer | 4,565 |  |  |  |
| No | 4,160 | 1.00 | (reference) |  |
| Yes | 405 | 1.45 | 1.20-1.76 | <0.001* |
| History of Liver disease | 4,565 |  |  |  |
| No | 3,797 | 1.00 | (reference) |  |
| Yes | 768 | 1.95 | 1.70-2.24 | <0.001* |
| History of Viral Hepatitis | 4,565 |  |  |  |
| No | 4,317 | 1.00 | (reference) |  |
| Yes | 248 | 1.01 | 0.76-1.34 | 0.946 |
| HIV | 4,565 |  |  |  |
| No | 4,400 | 1.00 | (reference) |  |
| Yes | 165 | 0.83 | 0.56-1.21 | 0.329 |
| Rheumatologic disease | 4,565 |  |  |  |
| No | 4,437 | 1.00 | (reference) |  |
| Yes | 128 | 1.07 | 0.74-1.56 | 0.713 |
| Dementia | 4,565 |  |  |  |
| No | 4,335 | 1.00 | (reference) |  |
| Yes | 230 | 1.43 | 1.12-1.82 | 0.004* |
| Paralysis | 4,565 |  |  |  |
| No | 4,447 | 1.00 | (reference) |  |
| Yes | 118 | 1.27 | 0.89-1.81 | 0.184 |
| Maximum level of respiratory support  (within first 24 hours of admission) | 4,565 |  |  |  |
| Room Air | 1,764 | 1.00 | (reference) |  |
| LFNC | 2,117 | 2.21 | 1.82-2.68 | <0.001* |
| HFNC | 523 | 6.77 | 5.61-8.17 | <0.001* |
| NIV | 161 | 3.06 | 2.19-4.26 | <0.001* |
| Remdesivir at least 1 day prior to lab collection | 4,565 |  |  |  |
| No | 4,519 | 1.00 | (reference) |  |
| Yes | 46 | 1.04 | 0.55-1.95 | 0.911 |
| AST | 4,286 |  |  |  |
| Normal | 2,024 | 1.00 | (reference) |  |
| Mild | 1,898 | 1.92 | 1.65-2.22 | <0.001* |
| Moderate | 321 | 2.34 | 1.88-2.91 | <0.001* |
| Severe | 43 | 2.43 | 1.48-4.00 | <0.001* |
| ALT | 4,297 |  |  |  |
| Normal | 2,750 | 1.00 | (reference) |  |
| Mild | 1,289 | 1.27 | 1.10-1.45 | 0.001* |
| Moderate | 235 | 1.32 | 1.01-1.72 | 0.040* |
| Severe | 23 | 1.37 | 0.63-3.00 | 0.424 |
| AP | 4,296 |  |  |  |
| Normal | 3,420 | 1.00 | (reference) |  |
| Elevated | 876 | 1.24 | 1.07-1.44 | 0.005* |
| Total Bilirubin | 4,297 |  |  |  |
| Normal | 3,838 | 1.00 | (reference) |  |
| Elevated | 459 | 1.19 | 0.98-1.44 | 0.081 |
| Direct Bilirubin | 447 |  |  |  |
| Normal | 34 | 1.00 | (reference) |  |
| Elevated | 413 | 2.50 | 0.83-7.48 | 0.102 |
| GGT | 42 |  |  |  |
| Normal | 13 | 1.00 | (reference) |  |
| Elevated | 29 | 0.90 | 0.26-3.09 | 0.863 |
| Albumin | 4,299 | 0.52 | 0.47-0.58 | <0.001* |
| Platelets | 4,552 | 0.998 | 0.997-0.999 | <0.001* |
| Creatinine | 4,556 | 1.01 | 0.99-1.04 | 0.195 |
| CRP | 3,144 | 1.03 | 1.03-1.04 | <0.001* |
| D-dimer | 2,749 | 1.01 | 1.00-1.02 | 0.039* |
| Hemoglobin | 4,552 | 1.03 | 1.01-1.06 | 0.019* |
| INR | 1,084 | 1.09 | 1.02-1.17 | 0.011* |
| PT | 1,084 | 1.01 | 1.00-1.01 | 0.077 |
| BUN | 4,554 | 1.01 | 1.01-1.01 | <0.001* |
| LDH | 554 | 1.00 | 1.00-1.00 | <0.001* |
| FIB-4 | 4,282 |  |  |  |
| Low | 1,323 | 1.00 | (reference) |  |
| Indeterminate | 1,555 | 2.07 | 1.67-2.56 | <0.001* |
| High | 1,404 | 3.35 | 2.74-4.09 | <0.001* |
| NFS | 4,238 |  |  |  |
| Low | 1,133 | 1.00 | (reference) |  |
| Indeterminate | 1,595 | 1.87 | 1.49-2.34 | <0.001* |
| High | 1,510 | 3.14 | 2.55-3.88 | <0.001* |
| APRI | 4,282 |  |  |  |
| Low | 2,174 | 1.00 | (reference) |  |
| Intermediate | 1,200 | 1.80 | 1.53-2.11 | <0.001* |
| High | 908 | 2.22 | 1.89-2.60 | <0.001* |
| De Ritis Ratio | 4,286 |  |  |  |
| <1 | 939 | 1.00 | (reference) |  |
| 1-2 | 2,431 | 1.53 | 1.25-1.87 | <0.001* |
| >2 | 916 | 2.21 | 1.78-2.73 | <0.001* |
| LFT Pattern | 4,295 |  |  |  |
| Mixed | 852 | 1.00 | (reference) |  |
| Hepatocellular | 164 | 1.09 | 0.78-1.52 | 0.607 |
| Cholestatic | 3,279 | 0.88 | 0.75-1.04 | 0.126 |

An asterisk (*) indicates statistical significance (p < 0.05). Abbreviations: RR=relative risk; CI=confidence interval; BMI=body mass index; CV=cardiovascular; HTN=hypertension; DM=diabetes mellitus; CKD=chronic kidney disease; HIV=human immunodeficiency virus; LFNC=low-flow nasal cannula; HFNC=high-flow nasal cannula; NIV=non-invasive ventilation; AST=aspartate aminotransferase; ALT=alanine aminotransferase; AP=alkaline phosphatase; GGT=gamma-glutamyl transferase; CRP=C-reactive protein; BUN=blood urea nitrogen; LDH=lactate dehydrogenase; INR=international normalized ratio; PT=prothrombin time; FIB-4=fibrosis-4 index; APRI=AST to platelet ratio index; NFS=non-alcoholic fatty liver disease fibrosis score; LFT=liver function test

## Table S3. LFTs and composite liver scores in the liver disease and non-liver disease subgroups.

|  | **Total** | **Liver Disease Subgroup** | **Non-liver Disease Subgroup** | **p-value^a^** |
| --- | --- | --- | --- | --- |
|  | **4,565 (100.0%)** | **956 (20.9%)** | **3,609 (79.1%)** |  |
| Severe COVID-19 | 766 (16.8%) | 248 (25.9%) | 518 (14.4%) | <0.001* |
| AST (U/L) | 42 (28-68) (N=4,286) | 55 (34-93) | 40 (27-62) | <0.001* |
| AST Elevation Severity |  |  |  |  |
| Normal | 2,024 (47.2%) | 306 (33.4%) | 1,718 (51.0%) | <0.001* |
| Mild | 1,898 (44.3%) | 454 (49.5%) | 1,444 (42.9%) |  |
| Moderate | 321 (7.5%) | 135 (14.7%) | 186 (5.5%) |  |
| Severe | 43 (1.0%) | 22 (2.4%) | 21 (0.6%) |  |
| ALT (U/L) | 30 (19-52) (N=4,297) | 36 (21-64) | 29 (18-49) | <0.001* |
| ALT Elevation Severity |  |  |  |  |
| Normal | 2,750 (64.0%) | 495 (53.8%) | 2,255 (66.8%) | <0.001* |
| Mild | 1,289 (30.0%) | 329 (35.8%) | 960 (28.4%) |  |
| Moderate | 235 (5.5%) | 79 (8.6%) | 156 (4.6%) |  |
| Severe | 23 (0.5%) | 17 (1.8%) | 6 (0.2%) |  |
| AP (U/L) | 91 (71-120) (N=4,296) | 105 (77-157) | 88 (70-114) | <0.001* |
| AP Elevation |  |  |  |  |
| Normal | 3,420 (79.6%) | 608 (66.2%) | 2,812 (83.3%) | <0.001* |
| Elevated | 876 (20.4%) | 311 (33.8%) | 565 (16.7%) |  |
| Total Bilirubin (mg/dL) | 0.5 (0.3-0.7) (N=4,297) | 0.6 (0.4-1.2) | 0.5 (0.3-0.7) | <0.001* |
| Total Bilirubin Elevation |  |  |  |  |
| Normal | 3,838 (89.3%) | 687 (74.7%) | 3,151 (93.3%) | <0.001* |
| Elevated | 459 (10.7%) | 233 (25.3%) | 226 (6.7%) |  |
| Direct Bilirubin (mg/dL) | 0.8 (0.5-1.3) (N=447) | 1.0 (0.6-2.3) | 0.5 (0.3-0.9) | <0.001* |
| Direct Bilirubin Elevation |  |  |  |  |
| Normal | 34 (7.6%) | 5 (2.2%) | 29 (13.2%) | <0.001* |
| Elevated | 413 (92.4%) | 222 (97.8%) | 191 (86.8%) |  |
| GGT (U/L) | 138 (37-254) (N=42) | 223 (65-322) | 73 (28-172) | 0.030* |
| GGT Elevation |  |  |  |  |
| Normal | 13 (31.0%) | 5 (22.7%) | 8 (40.0%) | 0.227 |
| Elevated | 29 (69.0%) | 17 (77.3%) | 12 (60.0%) |  |
| FIB-4 | 1.87 (1.11-3.22) (N=4,282) | 2.62 (1.45-5.50) | 1.75 (1.04-2.87) | <0.001* |
| FIB-4 Risk Categories |  |  |  |  |
| Low | 1,323 (30.9%) | 197 (21.5%) | 1,126 (33.4%) | <0.001* |
| Indeterminate | 1,555 (36.3%) | 268 (29.3%) | 1,287 (38.2%) |  |
| High | 1,404 (32.8%) | 450 (49.2%) | 954 (28.3%) |  |
| NFS | -0.09 (-1.58 - +1.35) (N=4,238) | +0.55 (-1.07 - +2.25) | -0.23 (-1.71 - +1.12) | <0.001* |
| NFS Risk Categories |  |  |  |  |
| Low | 1,133 (26.7%) | 179 (19.8%) | 954 (28.6%) | <0.001* |
| Indeterminate | 1,595 (37.6%) | 290 (32.1%) | 1,305 (39.1%) |  |
| High | 1,510 (35.6%) | 435 (48.1%) | 1,075 (32.2%) |  |
| APRI | 0.49 (0.28-0.88) (N=4,282) | 0.76 (0.39-1.59) | 0.44 (0.27-0.76) | <0.001* |
| APRI Risk Categories |  |  |  |  |
| Low | 2,174 (50.8%) | 301 (32.9%) | 1,873 (55.6%) | <0.001* |
| Intermediate | 1,200 (28.0%) | 249 (27.2%) | 951 (28.2%) |  |
| High | 908 (21.2%) | 365 (39.9%) | 543 (16.1%) |  |
| De Ritis Ratio | 1.40 (1.03-1.92) (N=4,286) | 1.56 (1.07-2.27) | 1.36 (1.01-1.84) | <0.001* |
| De Ritis Ratio Cutoff Categories |  |  |  |  |
| <1 | 939 (21.9%) | 183 (20.0%) | 756 (22.4%) | <0.001* |
| 1-2 | 2,431 (56.7%) | 440 (48.0%) | 1,991 (59.1%) |  |
| >2 | 916 (21.4%) | 294 (32.1%) | 622 (18.5%) |  |
| LFT Pattern |  |  |  |  |
| Mixed | 852 (19.8%) | 181 (19.7%) | 671 (19.9%) | <0.001* |
| Hepatocellular | 164 (3.8%) | 59 (6.4%) | 105 (3.1%) |  |
| Cholestatic | 3,279 (76.3%) | 679 (73.9%) | 2,600 (77.0%) |  |

^a^Mann-Whitney U test for continuous variables; Chi^2^ test for categorical variables

Note: Percentages may not add up to 100% due to rounding. Continuous variables are reported as median (interquartile range). Categorical variables are reported as N (%). An asterisk (*) indicates statistical significance (p < 0.05). Abbreviations: AST=aspartate aminotransferase; ALT=alanine aminotransferase; AP=alkaline phosphatase; GGT=gamma-glutamyl transferase; FIB-4=fibrosis-4 index; APRI=AST to platelet ratio index; NFS=non-alcoholic fatty liver disease fibrosis score; LFT=liver function test

## Table S4. Unadjusted relative risk of severe COVID-19 in the Non-Liver disease subgroup

|  | **N** | **RR** | **95% CI** | **p-value** |
| --- | --- | --- | --- | --- |
| Age | 3,609 | 1.02 | 1.02-1.03 | <0.001* |
| Sex | 3,609 |  |  |  |
| Female | 1,547 | 1.00 | (reference) |  |
| Male | 2,062 | 1.21 | 1.03-1.43 | 0.022* |
| Race | 3,609 |  |  |  |
| White | 2,599 | 1.00 | (reference) |  |
| Black | 874 | 0.58 | 0.46-0.73 | <0.001* |
| Asian | 68 | 1.11 | 0.66-1.86 | 0.707 |
| Native American/Alaska Native | 7 | 0.89 | 0.15-5.51 | 0.904 |
| Other Pacific Islander | 13 | 3E^-06^ | 2E^-06^-5E^-06^ | <0.001* |
| Unknown | 48 | 1.17 | 0.65-2.13 | 0.597 |
| Ethnicity | 3,609 |  |  |  |
| Not Hispanic | 1,363 | 1.00 | (reference) |  |
| Hispanic | 2,188 | 1.57 | 1.32-1.88 | <0.001* |
| Unknown | 58 | 0.48 | 0.16-1.47 | 0.200 |
| BMI | 3,577 | 1.01 | 1.00-1.02 | 0.020* |
| Previous CV complications | 3,609 |  |  |  |
| No | 2,785 | 1.00 | (reference) |  |
| Yes | 824 | 1.62 | 1.37-1.92 | <0.001* |
| HTN | 3,609 |  |  |  |
| No | 2,174 | 1.00 | (reference) |  |
| Yes | 1,435 | 1.09 | 0.93-1.28 | 0.284 |
| Respiratory disease | 3,609 |  |  |  |
| No | 2,944 | 1.00 | (reference) |  |
| Yes | 665 | 1.72 | 1.45-2.04 | <0.001* |
| DM | 3,609 |  |  |  |
| No | 1,889 | 1.00 | (reference) |  |
| Yes | 1,720 | 1.39 | 1.18-1.63 | <0.001* |
| CKD | 3,609 |  |  |  |
| No | 2,744 | 1.00 | (reference) |  |
| Yes | 865 | 1.34 | 1.13-1.59 | 0.001* |
| Cancer | 3,609 |  |  |  |
| No | 3,335 | 1.00 | (reference) |  |
| Yes | 274 | 1.48 | 1.15-1.89 | 0.002* |
| HIV | 3,609 |  |  |  |
| No | 3,500 | 1.00 | (reference) |  |
| Yes | 109 | 0.50 | 0.26-0.99 | 0.045* |
| Rheumatologic disease | 3,609 |  |  |  |
| No | 3,517 | 1.00 | (reference) |  |
| Yes | 92 | 0.98 | 0.59-1.64 | 0.951 |
| Dementia | 3,609 |  |  |  |
| No | 3,409 | 1.00 | (reference) |  |
| Yes | 200 | 1.74 | 1.34-2.26 | <0.001* |
| Paralysis | 3,609 |  |  |  |
| No | 3,505 | 1.00 | (reference) |  |
| Yes | 104 | 1.49 | 1.02-2.19 | 0.038* |
| Maximum level of respiratory support (within first 24 hours of admission) | 3,609 |  |  |  |
| Room Air | 1,350 | 1.00 | (reference) |  |
| LFNC | 1,729 | 2.27 | 1.77-2.91 | <0.001* |
| HFNC | 409 | 7.83 | 6.16-9.96 | <0.001* |
| NIV | 121 | 4.01 | 2.71-5.91 | <0.001* |
| AST | 3,369 |  |  |  |
| Normal | 1,718 | 1.00 | (reference) |  |
| Mild | 1,444 | 1.97 | 1.65-2.35 | <0.001* |
| Moderate | 186 | 2.42 | 1.81-3.23 | <0.001* |
| Severe | 21 | 1.43 | 0.50-4.11 | 0.510 |
| ALT | 3,377 |  |  |  |
| Normal | 2,255 | 1.00 | (reference) |  |
| Mild | 960 | 1.29 | 1.09-1.53 | 0.004* |
| Moderate | 156 | 1.12 | 0.76-1.64 | 0.564 |
| Severe | 6 | 1.21 | 0.20-7.28 | 0.833 |
| AP | 3,377 |  |  |  |
| Normal | 2,812 | 1.00 | (reference) |  |
| Elevated | 565 | 1.32 | 1.09-1.61 | 0.005* |
| Total bilirubin | 3,377 |  |  |  |
| Normal | 3,151 | 1.00 | (reference) |  |
| Elevated | 226 | 1.13 | 0.84-1.53 | 0.413 |
| Direct bilirubin | 220 |  |  |  |
| Normal | 29 | 1.00 | (reference) |  |
| Elevated | 191 | 5.47 | 0.78-38.52 | 0.088 |
| GGT | 20 |  |  |  |
| Normal | 8 | 1.00 | (reference) |  |
| Elevated | 12 | 2.00 | 0.24-16.88 | 0.524 |
| Albumin | 3,379 | 0.46 | 0.40-0.52 | <0.001* |
| Platelets | 3,598 | 0.998 | 0.997-0.999 | <0.001* |
| Creatinine | 3,600 | 1.01 | 0.99-1.04 | 0.404 |
| CRP | 2,547 | 1.04 | 1.03-1.05 | <0.001* |
| D-dimer | 2,224 | 1.02 | 1.00-1.03 | 0.016* |
| Hemoglobin | 3,600 | 1.04 | 1.00-1.07 | 0.036* |
| INR | 761 | 1.04 | 0.92-1.18 | 0.510 |
| PT | 761 | 1.00 | 0.99-1.01 | 0.738 |
| BUN | 3,598 | 1.01 | 1.01-1.01 | <0.001* |
| LDH | 444 | 1.00 | 1.00-1.00 | 0.088 |
| FIB-4 | 3,367 |  |  |  |
| Low | 1,126 | 1.00 | (reference) |  |
| Indeterminate | 1,287 | 2.25 | 1.74-2.90 | <0.001* |
| High | 954 | 3.84 | 3.01-4.92 | <0.001* |
| NFS | 3,334 |  |  |  |
| Low | 954 | 1.00 | (reference) |  |
| Indeterminate | 1,305 | 1.85 | 1.42-2.42 | <0.001* |
| High | 1,075 | 3.33 | 2.59-4.28 | <0.001* |
| APRI | 3,367 |  |  |  |
| Low | 1,873 | 1.00 | (reference) |  |
| Intermediate | 951 | 1.82 | 1.51-2.19 | <0.001* |
| High | 543 | 2.25 | 1.84-2.75 | <0.001* |
| De Ritis ratio | 3,369 |  |  |  |
| <1 | 756 | 1.00 | (reference) |  |
| 1-2 | 1,991 | 1.52 | 1.20-1.93 | 0.001* |
| >2 | 622 | 2.11 | 1.62-2.74 | <0.001* |
| LFT pattern | 3,376 |  |  |  |
| Mixed | 671 | 1.00 | (reference) |  |
| Hepatocellular | 105 | 0.73 | 0.43-1.25 | 0.247 |
| Cholestatic | 2,600 | 0.86 | 0.71-1.04 | 0.111 |

An asterisk (*) indicates statistical significance (p < 0.05). Abbreviations: RR=relative risk; CI=confidence interval; BMI=body mass index; CV=cardiovascular; HTN=hypertension; DM=diabetes mellitus; CKD=chronic kidney disease; HIV=human immunodeficiency virus; LFNC=low-flow nasal cannula; HFNC=high-flow nasal cannula; NIV=non-invasive ventilation; AST=aspartate aminotransferase; ALT=alanine aminotransferase; AP=alkaline phosphatase; GGT=gamma-glutamyl transferase; CRP=C-reactive protein; BUN=blood urea nitrogen; LDH=lactate dehydrogenase; INR=international normalized ratio; PT=prothrombin time; FIB-4=fibrosis-4 index; APRI=AST to platelet ratio index; NFS=non-alcoholic fatty liver disease fibrosis score; LFT=liver function test

## Table S5. LFTs and composite liver scores in the oxygen support and room air subgroups.

|  | **Total** | **Oxygen Support Subgroup** | **Room Air Subgroup** | **p-value^a^** |
| --- | --- | --- | --- | --- |
|  | **4,565 (100.0%)** | **2,801 (61.4%)** | **1,764 (38.6%)** |  |
| Severe COVID-19 | 766 (16.8%) | 637 (22.7%) | 129 (7.3%) | <0.001* |
| AST (U/L) | 42 (28-68) (N=4,286) | 48 (33-74) | 33 (22-53) | <0.001* |
| AST Elevation Severity |  |  |  |  |
| Normal | 2,024 (47.2%) | 1,042 (38.6%) | 982 (61.8%) | <0.001* |
| Mild | 1,898 (44.3%) | 1,422 (52.7%) | 476 (30.0%) |  |
| Moderate | 321 (7.5%) | 217 (8.0%) | 104 (6.5%) |  |
| Severe | 43 (1.0%) | 17 (0.6%) | 26 (1.6%) |  |
| ALT (U/L) | 30 (19-52) (N=4,297) | 35 (21-57) | 24 (16-41) | <0.001* |
| ALT Elevation Severity |  |  |  |  |
| Normal | 2,750 (64.0%) | 1,562 (57.8%) | 1,188 (74.4%) | <0.001* |
| Mild | 1,289 (30.0%) | 964 (35.7%) | 325 (20.4%) |  |
| Moderate | 235 (5.5%) | 167 (6.2%) | 68 (4.3%) |  |
| Severe | 23 (0.5%) | 8 (0.3%) | 15 (0.9%) |  |
| AP (U/L) | 91 (71-120) (N=4,296) | 87 (69-115) | 99 (76-134) | <0.001* |
| AP Elevation |  |  |  |  |
| Normal | 3,420 (79.6%) | 2,239 (83.0%) | 1,181 (74.0%) | <0.001* |
| Elevated | 876 (20.4%) | 460 (17.0%) | 416 (26.0%) |  |
| Total Bilirubin (mg/dL) | 0.5 (0.3-0.7) (N=4,297) | 0.5 (0.3-0.7) | 0.5 (0.3-0.8) | 0.098 |
| Total Bilirubin Elevation |  |  |  |  |
| Normal | 3,838 (89.3%) | 2,490 (92.2%) | 1,348 (84.5%) | <0.001* |
| Elevated | 459 (10.7%) | 212 (7.8%) | 247 (15.5%) |  |
| Direct Bilirubin (mg/dL) | 0.8 (0.5-1.3) (N=447) | 0.8 (0.5-1.2) | 0.7 (0.4-1.9) | 0.453 |
| Direct Bilirubin Elevation |  |  |  |  |
| Normal | 34 (7.6%) | 19 (8.8%) | 15 (6.5%) | 0.373 |
| Elevated | 413 (92.4%) | 198 (91.2%) | 215 (93.5%) |  |
| GGT (U/L) | 138 (37-254) (N=42) | 125 (53-262) | 152 (31-254) | 0.636 |
| GGT Elevation |  |  |  |  |
| Normal | 13 (31.0%) | 2 (18.2%) | 11 (35.5%) | 0.286 |
| Elevated | 29 (69.0%) | 9 (81.8%) | 20 (64.5%) |  |
| FIB-4 | 1.87 (1.11-3.22) (N=4,282) | 2.03 (1.30-3.24) | 1.58 (0.87-3.16) | <0.001* |
| FIB-4 Risk Categories |  |  |  |  |
| Low | 1,323 (30.9%) | 675 (25.0%) | 648 (40.9%) | <0.001* |
| Indeterminate | 1,555 (36.3%) | 1,098 (40.7%) | 457 (28.8%) |  |
| High | 1,404 (32.8%) | 924 (34.3%) | 480 (30.3%) |  |
| NFS | -0.09 (-1.58 - +1.35) (N=4,238) | +0.15 (-1.25 - +1.54) | -0.55 (-2.23 - +1.03) | <0.001* |
| NFS Risk Categories |  |  |  |  |
| Low | 1,133 (26.7%) | 579 (21.7%) | 554 (35.4%) | <0.001* |
| Indeterminate | 1,595 (37.6%) | 1,047 (39.2%) | 548 (35.0%) |  |
| High | 1,510 (35.6%) | 1,046 (39.1%) | 464 (29.6%) |  |
| APRI | 0.49 (0.28-0.88) (N=4,282) | 0.56 (0.35-0.93) | 0.37 (0.20-0.76) | <0.001* |
| APRI Risk Categories |  |  |  |  |
| Low | 2,174 (50.8%) | 1,196 (44.3%) | 978 (61.7%) | <0.001* |
| Intermediate | 1,200 (28.0%) | 898 (33.3%) | 302 (19.1%) |  |
| High | 908 (21.2%) | 603 (22.4%) | 305 (19.2%) |  |
| De Ritis Ratio | 1.40 (1.03-1.92) (N=4,286) | 1.39 (1.04-1.90) | 1.40 (1.00-1.93) | 0.858 |
| De Ritis Ratio Cutoff Categories |  |  |  |  |
| <1 | 939 (21.9%) | 568 (21.1%) | 371 (23.4%) | 0.066 |
| 1-2 | 2,431 (56.7%) | 1,566 (58.0%) | 865 (54.5%) |  |
| >2 | 916 (21.4%) | 564 (20.9%) | 352 (22.2%) |  |
| LFT Pattern |  |  |  |  |
| Mixed | 852 (19.8%) | 669 (24.8%) | 183 (11.5%) | <0.001* |
| Hepatocellular | 164 (3.8%) | 120 (4.4%) | 44 (2.8%) |  |
| Cholestatic | 3,279 (76.3%) | 1,910 (70.8%) | 1,369 (85.8%) |  |

^a^Mann-Whitney U test for continuous variables; Chi^2^ test for categorical variables

Note: Percentages may not add up to 100% due to rounding. Continuous variables are reported as median (interquartile range). Categorical variables are reported as N (%). An asterisk (*) indicates statistical significance (p < 0.05). Abbreviations: AST=aspartate aminotransferase; ALT=alanine aminotransferase; AP=alkaline phosphatase; GGT=gamma-glutamyl transferase; FIB-4=fibrosis-4 index; APRI=AST to platelet ratio index; NFS=non-alcoholic fatty liver disease fibrosis score; LFT=liver function test

## Table S6. Unadjusted relative risks of severe COVID-19 in the room air subgroup

|  | **N** | **RR** | **95% CI** | **p-value** |
| --- | --- | --- | --- | --- |
| Age | 1,764 | 1.02 | 1.01-1.03 | <0.001* |
| Sex | 1,764 |  |  |  |
| Female | 710 | 1.00 | (reference) |  |
| Male | 1,054 | 1.14 | 0.81-1.60 | 0.465 |
| Race | 1,764 |  |  |  |
| White | 1,177 | 1.00 | (reference) |  |
| Black | 522 | 0.82 | 0.55-1.21 | 0.320 |
| Asian | 35 | 2.68 | 1.34-5.35 | 0.005* |
| Native American/Alaska Native | 4 | 3.34 | 0.61-18.48 | 0.166 |
| Other Pacific Islander | 5 | 7E^-6^ | 3E^-6^-2E^-5^ | <0.001* |
| Unknown | 21 | 0.64 | 0.09-4.36 | 0.646 |
| Ethnicity | 1,764 |  |  |  |
| Not Hispanic | 816 | 1.00 | (reference) |  |
| Hispanic | 916 | 1.03 | 0.73-1.44 | 0.878 |
| Unknown | 32 | 0.86 | 0.22-3.38 | 0.834 |
| BMI | 1,747 | 1.00 | 0.98-1.03 | 0.794 |
| Previous CV complications | 1,764 |  |  |  |
| No | 1,326 | 1.00 | (reference) |  |
| Yes | 438 | 1.92 | 1.37-2.69 | <0.001* |
| HTN | 1,764 |  |  |  |
| No | 1,035 | 1.00 | (reference) |  |
| Yes | 729 | 0.79 | 0.56-1.11 | 0.177 |
| Respiratory disease | 1,764 |  |  |  |
| No | 1,451 | 1.00 | (reference) |  |
| Yes | 313 | 2.01 | 1.41-2.87 | <0.001* |
| DM | 1,764 |  |  |  |
| No | 955 | 1.00 | (reference) |  |
| Yes | 809 | 1.06 | 0.76-1.48 | 0.736 |
| CKD | 1,764 |  |  |  |
| No | 1,268 | 1.00 | (reference) |  |
| Yes | 496 | 1.67 | 1.19-2.34 | 0.003* |
| Cancer | 1,764 |  |  |  |
| No | 1,532 | 1.00 | (reference) |  |
| Yes | 232 | 2.36 | 1.64-3.41 | <0.001* |
| History of liver disease | 1,764 |  |  |  |
| No | 1,434 | 1.00 | (reference) |  |
| Yes | 330 | 2.02 | 1.43-2.88 | <0.001* |
| History of viral hepatitis | 1,764 |  |  |  |
| No | 1,624 | 1.00 | (reference) |  |
| Yes | 140 | 1.53 | 0.92-2.54 | 0.104 |
| HIV | 1,764 |  |  |  |
| No | 1,664 | 1.00 | (reference) |  |
| Yes | 100 | 1.10 | 0.55-2.19 | 0.785 |
| Rheumatologic disease | 1,764 |  |  |  |
| No | 1,716 | 1.00 | (reference) |  |
| Yes | 48 | 0.56 | 0.14-2.21 | 0.410 |
| Dementia | 1,764 |  |  |  |
| No | 1,653 | 1.00 | (reference) |  |
| Yes | 111 | 1.96 | 1.18-3.24 | 0.009* |
| Paralysis | 1,764 |  |  |  |
| No | 1,686 | 1.00 | (reference) |  |
| Yes | 78 | 1.62 | 0.86-3.07 | 0.138 |
| Remdesivir at least 1 day prior to lab collection | 1,764 |  |  |  |
| No | 1,762 | 1.00 | (reference) |  |
| Yes | 2 | 7E^-9^ | 2E^-9^-3E^-8^ | <0.001* |
| AST | 1,588 |  |  |  |
| Normal | 982 | 1.00 | (reference) |  |
| Mild | 476 | 1.51 | 1.04-2.20 | 0.030* |
| Moderate | 104 | 1.73 | 0.94-3.19 | 0.078 |
| Severe | 26 | 3.15 | 1.38-7.18 | 0.006* |
| ALT | 1,596 |  |  |  |
| Normal | 1,188 | 1.00 | (reference) |  |
| Mild | 325 | 1.23 | 0.82-1.86 | 0.317 |
| Moderate | 68 | 1.26 | 0.57-2.79 | 0.563 |
| Severe | 15 | 2.86 | 1.02-8.05 | 0.046* |
| AP | 1,597 |  |  |  |
| Normal | 1,181 | 1.00 | (reference) |  |
| Elevated | 416 | 1.53 | 1.07-2.19 | 0.020* |
| Total bilirubin | 1,595 |  |  |  |
| Normal | 1,348 | 1.00 | (reference) |  |
| Elevated | 247 | 1.98 | 1.36-2.91 | <0.001* |
| Direct bilirubin | 230 |  |  |  |
| Normal | 15 | 1.00 | (reference) |  |
| Elevated | 215 | 2573226 | 1306803-5066941 | <0.001* |
| GGT | 31 |  |  |  |
| Normal | 11 | 1.00 | (reference) |  |
| Elevated | 20 | 0.82 | 0.16-4.33 | 0.820 |
| Albumin | 1,595 | 0.48 | 0.38-0.60 | <0.001* |
| Platelets | 1,753 | 0.997 | 0.995-0.998 | <0.001* |
| Creatinine | 1,756 | 1.06 | 1.03-1.10 | 0.001* |
| CRP | 804 | 1.03 | 1.01-1.05 | 0.006* |
| D-dimer | 597 | 1.01 | 0.96-1.06 | 0.680 |
| Hemoglobin | 1,754 | 0.90 | 0.85-0.96 | 0.001* |
| INR | 563 | 1.20 | 0.99-1.45 | 0.061 |
| PT | 563 | 1.02 | 1.00-1.03 | 0.062 |
| BUN | 1,754 | 1.01 | 1.01-1.02 | <0.001* |
| LDH | 172 | 1.00 | 1.00-1.00 | 0.659 |
| FIB-4 | 1,585 |  |  |  |
| Low | 648 | 1.00 | (reference) |  |
| Indeterminate | 457 | 1.27 | 0.76-2.13 | 0.362 |
| High | 480 | 3.03 | 1.98-4.61 | <0.001* |
| NFS | 1,566 |  |  |  |
| Low | 554 | 1.00 | (reference) |  |
| Indeterminate | 548 | 2.18 | 1.28-3.71 | 0.004* |
| High | 464 | 3.77 | 2.28-6.22 | <0.001* |
| APRI | 1,585 |  |  |  |
| Low | 978 | 1.00 | (reference) |  |
| Intermediate | 302 | 1.50 | 0.95-2.37 | 0.082 |
| High | 305 | 2.43 | 1.66-3.58 | <0.001* |
| De Ritis Ratio | 1,588 |  |  |  |
| <1 | 371 | 1.00 | (reference) |  |
| 1-2 | 865 | 1.82 | 1.07-3.10 | 0.027* |
| >2 | 352 | 2.37 | 1.34-4.20 | 0.003* |
| LFT pattern | 1,596 |  |  |  |
| Mixed | 183 | 1.00 | (reference) |  |
| Hepatocellular | 44 | 1.39 | 0.53-3.61 | 0.504 |
| Cholestatic | 1,369 | 0.89 | 0.53-1.50 | 0.664 |

An asterisk (*) indicates statistical significance (p < 0.05). Abbreviations: RR=relative risk; CI=confidence interval; BMI=body mass index; CV=cardiovascular; HTN=hypertension; DM=diabetes mellitus; CKD=chronic kidney disease; HIV=human immunodeficiency virus; LFNC=low-flow nasal cannula; HFNC=high-flow nasal cannula; NIV=non-invasive ventilation; AST=aspartate aminotransferase; ALT=alanine aminotransferase; AP=alkaline phosphatase; GGT=gamma-glutamyl transferase; CRP=C-reactive protein; BUN=blood urea nitrogen; LDH=lactate dehydrogenase; INR=international normalized ratio; PT=prothrombin time; FIB-4=fibrosis-4 index; APRI=AST to platelet ratio index; NFS=non-alcoholic fatty liver disease fibrosis score; LFT=liver function test

## Table S7. LFTs and composite liver scores by variant-predominant period

|  | **Total** | **Pre-Delta** | **Delta** | **Omicron** | **p-value^a^** |
| --- | --- | --- | --- | --- | --- |
|  | **4,565 (100.0%)** | **2,293 (50.2%)** | **820 (18.0%)** | **1,452 (31.8%)** |  |
| Severe COVID-19 | 766 (16.8%) | 428 (18.7%) | 176 (21.5%) | 162 (11.2%) | <0.001* |
| AST (U/L) | 42 (28-68) (N=4,286) | 44 (30-68) | 52 (34-82) | 34 (23-56) | <0.001* |
| AST Elevation Severity |  |  |  |  |  |
| Normal | 2,024 (47.2%) | 961 (44.3%) | 274 (35.4%) | 789 (58.8%) | <0.001* |
| Mild | 1,898 (44.3%) | 1,054 (48.5%) | 399 (51.6%) | 445 (33.2%) |  |
| Moderate | 321 (7.5%) | 139 (6.4%) | 90 (11.6%) | 92 (6.9%) |  |
| Severe | 43 (1.0%) | 17 (0.8%) | 10 (1.3%) | 16 (1.2%) |  |
| ALT (U/L) | 30 (19-52) (N=4,297) | 32 (20-53) | 38 (22-64) | 24 (15-42) | <0.001* |
| ALT Elevation Severity |  |  |  |  |  |
| Normal | 2,750 (64.0%) | 1,353 (62.2%) | 410 (52.8%) | 987 (73.3%) | <0.001* |
| Mild | 1,289 (30.0%) | 696 (32.0%) | 302 (38.9%) | 291 (21.6%) |  |
| Moderate | 235 (5.5%) | 119 (5.5%) | 59 (7.6%) | 57 (4.2%) |  |
| Severe | 23 (0.5%) | 6 (0.3%) | 5 (0.6%) | 12 (0.9%) |  |
| AP (U/L) | 91 (71-120) (N=4,296) | 88 (70-118) | 86 (68-113) | 98 (75-131) | <0.001* |
| AP Elevation |  |  |  |  |  |
| Normal | 3,420 (79.6%) | 1,767 (81.4%) | 646 (83.2%) | 1,007 (74.7%) | <0.001* |
| Elevated | 876 (20.4%) | 405 (18.6%) | 130 (16.8%) | 341 (25.3%) |  |
| Total Bilirubin (mg/dL) | 0.5 (0.3-0.7) (N=4,297) | 0.5 (0.3-0.7) | 0.5 (0.3-0.7) | 0.5 (0.3-0.8) | 0.095 |
| Total Bilirubin Elevation |  |  |  |  |  |
| Normal | 3,838 (89.3%) | 1,980 (91.2%) | 719 (92.5%) | 1,139 (84.5%) | <0.001* |
| Elevated | 459 (10.7%) | 192 (8.8%) | 58 (7.5%) | 209 (15.5%) |  |
| Direct Bilirubin (mg/dL) | 0.8 (0.5-1.3) (N=447) | 0.7 (0.4-1.2) | 0.9 (0.5-1.2) | 0.8 (0.5-1.5) | 0.139 |
| Direct Bilirubin Elevation |  |  |  |  |  |
| Normal | 34 (7.6%) | 21 (11.2%) | 3 (5.3%) | 10 (5.0%) | 0.053 |
| Elevated | 413 (92.4%) | 167 (88.8%) | 54 (94.7%) | 192 (95.0%) |  |
| GGT (U/L) | 138 (37-254) (N=42) | 58 (31-219) | 360 (74-646) | 172 (74-292) | 0.177 |
| GGT Elevation |  |  |  |  |  |
| Normal | 13 (31.0%) | 8 (44.4%) | 0 (0.0%) | 5 (25.0%) | 0.161 |
| Elevated | 29 (69.0%) | 10 (55.6%) | 4 (100.0%) | 15 (75.0%) |  |
| FIB-4 | 1.87 (1.11-3.22) (N=4,282) | 1.92 (1.19-3.24) | 1.82 (1.12-3.01) | 1.81 (1.01-3.28) | 0.081 |
| FIB-4 Risk Categories |  |  |  |  |  |
| Low | 1,323 (30.9%) | 628 (29.0%) | 242 (31.4%) | 453 (33.8%) | 0.006* |
| Indeterminate | 1,555 (36.3%) | 813 (37.5%) | 298 (38.7%) | 444 (33.1%) |  |
| High | 1,404 (32.8%) | 728 (33.6%) | 231 (30.0%) | 445 (33.2%) |  |
| NFS | -0.09 (-1.58 - +1.35) (N=4,238) | +0.04 (-1.36 - +1.46) | -0.45 (-1.79 - +1.12) | -0.18 (-1.84 - +1.26) | <0.001* |
| NFS Risk Categories |  |  |  |  |  |
| Low | 1,133 (26.7%) | 505 (23.5%) | 232 (30.3%) | 396 (29.9%) | <0.001* |
| Indeterminate | 1,595 (37.6%) | 825 (38.4%) | 292 (38.2%) | 478 (36.1%) |  |
| High | 1,510 (35.6%) | 818 (38.1%) | 241 (31.5%) | 451 (34.0%) |  |
| APRI | 0.49 (0.28-0.88) (N=4,282) | 0.51 (0.31-0.88) | 0.60 (0.34-1.02) | 0.39 (0.22-0.78) | <0.001* |
| APRI Risk Categories |  |  |  |  |  |
| Low | 2,174 (50.8%) | 1,049 (48.4%) | 325 (42.2%) | 800 (59.6%) | <0.001* |
| Intermediate | 1,200 (28.0%) | 657 (30.3%) | 252 (32.7%) | 291 (21.7%) |  |
| High | 908 (21.2%) | 463 (21.3%) | 194 (25.2%) | 251 (18.7%) |  |
| De Ritis Ratio | 1.40 (1.03-1.92) (N=4,286) | 1.36 (1.03-1.88) | 1.38 (1.00-1.88) | 1.46 (1.05-2.00) | 0.003* |
| De Ritis Ratio Cutoff Categories |  |  |  |  |  |
| <1 | 939 (21.9%) | 472 (21.7%) | 189 (24.5%) | 278 (20.7%) | 0.003* |
| 1-2 | 2,431 (56.7%) | 1,271 (58.5%) | 428 (55.4%) | 732 (54.5%) |  |
| >2 | 916 (21.4%) | 428 (19.7%) | 156 (20.2%) | 332 (24.7%) |  |
| LFT Pattern |  |  |  |  |  |
| Mixed | 852 (19.8%) | 478 (22.0%) | 216 (27.8%) | 158 (11.7%) | <0.001* |
| Hepatocellular | 164 (3.8%) | 80 (3.7%) | 48 (6.2%) | 36 (2.7%) |  |
| Cholestatic | 3,279 (76.3%) | 1,614 (74.3%) | 512 (66.0%) | 1,153 (85.6%) |  |

^a^Kruskal-Wallis test for continuous variables; Chi^2^ test for categorical variables

Note: Percentages may not add up to 100% due to rounding. Continuous variables are reported as median (interquartile range). Categorical variables are reported as N (%). An asterisk (*) indicates statistical significance (p < 0.05). Abbreviations: AST=aspartate aminotransferase; ALT=alanine aminotransferase; AP=alkaline phosphatase; GGT=gamma-glutamyl transferase; FIB-4=fibrosis-4 index; APRI=AST to platelet ratio index; NFS=non-alcoholic fatty liver disease fibrosis score; LFT=liver function test

## Table S8. Unadjusted relative risks of severe COVID-19 by Variant-predominant Era

|  | **Pre-Delta** | | | | **Delta** | | | | **Omicron** | | | |
| --- | --- | --- | --- | --- | --- | --- | --- | --- | --- | --- | --- | --- |
|  | **N** | **RR** | **95% CI** | **p-value** | **N** | **RR** | **95% CI** | **p-value** | **N** | **RR** | **95% CI** | **p-value** |
| Age | 2,293 | 1.03 | 1.02-1.03 | <0.001* | 820 | 1.02 | 1.01-1.02 | <0.001* | 1,452 | 1.02 | 1.01-1.03 | <0.001* |
| Sex | 2,293 |  |  |  | 820 |  |  |  | 1,452 |  |  |  |
| Female | 982 | 1.00 | (reference) |  | 318 | 1.00 | (reference) |  | 594 | 1.00 | (reference) |  |
| Male | 1,311 | 1.22 | 1.02-1.45 | 0.029* | 502 | 1.16 | 0.88-1.53 | 0.278 | 858 | 1.12 | 0.83-1.51 | 0.470 |
| Race | 2,293 |  |  |  | 820 |  |  |  | 1,452 |  |  |  |
| White | 1,773 | 1.00 | (reference) |  | 578 | 1.00 | (reference) |  | 929 | 1.00 | (reference) |  |
| Black | 430 | 0.55 | 0.41-0.72 | <0.001* | 222 | 0.66 | 0.47-0.93 | 0.016* | 459 | 0.85 | 0.61-1.18 | 0.338 |
| Asian | 38 | 1.29 | 0.75-2.21 | 0.357 | 9 | 2E^-6^ | 9E^-7^-3E^-6^ | <0.001* | 41 | 1.69 | 0.89-3.24 | 0.110 |
| Native American/Alaska Native | 5 | 1.96 | 0.67-5.76 | 0.221 | 3 | 1.40 | 0.28-6.97 | 0.684 | 3 | 6E^-6^ | 2E^-6^-2E^-5^ | <0.001* |
| Other Pacific Islander | 12 | 0.41 | 0.06-2.67 | 0.350 | 0 |  | No observations |  | 2 | 6E^-6^ | 1E^-6^-2E^-5^ | <0.001* |
| Unknown | 35 | 0.70 | 0.31-1.58 | 0.392 | 8 | 1.05 | 0.31-3.51 | 0.941 | 18 | 0.96 | 0.26-3.61 | 0.957 |
| Ethnicity | 2,293 |  |  |  | 820 |  |  |  | 1,452 |  |  |  |
| Not Hispanic | 707 | 1.00 | (reference) |  | 315 | 1.00 | (reference) |  | 727 | 1.00 | (reference) |  |
| Hispanic | 1,551 | 1.43 | 1.17-1.76 | 0.001* | 494 | 1.72 | 1.27-2.33 | <0.001* | 703 | 1.09 | 0.81-1.46 | 0.575 |
| Unknown | 35 | 0.20 | 0.03-1.37 | 0.100 | 11 | 1.22 | 0.34-4.39 | 0.762 | 22 | 0.85 | 0.22-3.23 | 0.808 |
| BMI | 2,274 | 1.01 | 1.00-1.02 | 0.011* | 814 | 1.02 | 1.01-1.03 | <0.001* | 1,435 | 0.99 | 0.97-1.00 | 0.146 |
| Previous CV complications | 2,293 |  |  |  | 820 |  |  |  | 1,452 |  |  |  |
| No | 1,820 | 1.00 | (reference) |  | 684 | 1.00 | (reference) |  | 939 | 1.00 | (reference) |  |
| Yes | 473 | 1.52 | 1.26-1.82 | <0.001* | 136 | 1.48 | 1.09-2.00 | 0.011* | 513 | 1.97 | 1.48-2.63 | <0.001* |
| HTN | 2,293 |  |  |  | 820 |  |  |  | 1,452 |  |  |  |
| No | 1,366 | 1.00 | (reference) |  | 501 | 1.00 | (reference) |  | 841 | 1.00 | (reference) |  |
| Yes | 927 | 1.08 | 0.91-1.28 | 0.384 | 319 | 1.17 | 0.90-1.52 | 0.253 | 611 | 0.81 | 0.60-1.09 | 0.170 |
| Respiratory disease | 2,293 |  |  |  | 820 |  |  |  | 1,452 |  |  |  |
| No | 1,924 | 1.00 | (reference) |  | 685 | 1.00 | (reference) |  | 1,028 | 1.00 | (reference) |  |
| Yes | 369 | 1.76 | 1.46-2.12 | <0.001* | 135 | 1.64 | 1.23-2.19 | 0.001* | 424 | 1.80 | 1.35-2.40 | <0.001* |
| DM | 2,293 |  |  |  | 820 |  |  |  | 1,452 |  |  |  |
| No | 1,123 | 1.00 | (reference) |  | 457 | 1.00 | (reference) |  | 784 | 1.00 | (reference) |  |
| Yes | 1,170 | 1.32 | 1.11-1.57 | 0.002* | 363 | 1.70 | 1.30-2.21 | <0.001* | 668 | 0.87 | 0.65-1.17 | 0.356 |
| CKD | 2,293 |  |  |  | 820 |  |  |  | 1,452 |  |  |  |
| No | 1,766 | 1.00 | (reference) |  | 673 | 1.00 | (reference) |  | 984 | 1.00 | (reference) |  |
| Yes | 527 | 1.45 | 1.21-1.73 | <0.001* | 147 | 1.43 | 1.07-1.93 | 0.017* | 468 | 1.20 | 0.89-1.63 | 0.225 |
| Cancer | 2,293 |  |  |  | 820 |  |  |  | 1,452 |  |  |  |
| No | 2,131 | 1.00 | (reference) |  | 774 | 1.00 | (reference) |  | 1,255 | 1.00 | (reference) |  |
| Yes | 162 | 1.43 | 1.09-1.89 | 0.011* | 46 | 1.12 | 0.66-1.91 | 0.673 | 197 | 2.23 | 1.62-3.06 | <0.001* |
| History of liver disease | 2,293 |  |  |  | 820 |  |  |  | 1,452 |  |  |  |
| No | 1,922 | 1.00 | (reference) |  | 720 | 1.00 | (reference) |  | 1,155 | 1.00 | (reference) |  |
| Yes | 371 | 2.09 | 1.75-2.49 | <0.001* | 100 | 2.55 | 1.96-3.32 | <0.001* | 297 | 1.64 | 1.20-2.24 | 0.002* |
| History of viral hepatitis | 2,293 |  |  |  | 820 |  |  |  | 1,452 |  |  |  |
| No | 2,216 | 1.00 | (reference) |  | 780 | 1.00 | (reference) |  | 1,321 | 1.00 | (reference) |  |
| Yes | 77 | 0.83 | 0.49-1.41 | 0.489 | 40 | 1.43 | 0.87-2.34 | 0.157 | 131 | 1.26 | 0.80-1.99 | 0.320 |
| HIV | 2,293 |  |  |  | 820 |  |  |  | 1,452 |  |  |  |
| No | 2,250 | 1.00 | (reference) |  | 796 | 1.00 | (reference) |  | 1,354 | 1.00 | (reference) |  |
| Yes | 43 | 0.62 | 0.27-1.42 | 0.256 | 24 | 0.77 | 0.31-1.91 | 0.574 | 98 | 1.31 | 0.79-2.17 | 0.302 |
| Rheumatologic disease | 2,293 |  |  |  | 820 |  |  |  | 1,452 |  |  |  |
| No | 2,241 | 1.00 | (reference) |  | 805 | 1.00 | (reference) |  | 1,391 | 1.00 | (reference) |  |
| Yes | 52 | 1.24 | 0.75-2.06 | 0.397 | 15 | 0.93 | 0.34-2.58 | 0.890 | 61 | 1.18 | 0.61-2.30 | 0.617 |
| Dementia | 2,293 |  |  |  | 820 |  |  |  | 1,452 |  |  |  |
| No | 2,197 | 1.00 | (reference) |  | 792 | 1.00 | (reference) |  | 1,346 | 1.00 | (reference) |  |
| Yes | 96 | 1.66 | 1.21-2.28 | 0.002* | 28 | 1.17 | 0.61-2.26 | 0.636 | 106 | 1.59 | 1.01-2.49 | 0.043* |
| Paralysis | 2,293 |  |  |  | 820 |  |  |  | 1,452 |  |  |  |
| No | 2,251 | 1.00 | (reference) |  | 806 | 1.00 | (reference) |  | 1,390 | 1.00 | (reference) |  |
| Yes | 42 | 1.28 | 0.74-2.22 | 0.374 | 14 | 1.34 | 0.58-3.10 | 0.496 | 62 | 1.63 | 0.94-2.85 | 0.084 |
| Maximum level of respiratory support  (within first 24 hours of admission) | 2,293 |  |  |  | 820 |  |  |  | 1,452 |  |  |  |
| Room Air | 741 | 1.00 | (reference) |  | 220 | 1.00 | (reference) |  | 803 | 1.00 | (reference) |  |
| LFNC | 1,185 | 2.25 | 1.69-2.99 | <0.001* | 438 | 3.61 | 1.96-6.64 | <0.001* | 494 | 1.68 | 1.21-2.33 | 0.002* |
| HFNC | 308 | 6.91 | 5.24-9.11 | <0.001* | 152 | 10.92 | 6.03-19.79 | <0.001* | 63 | 3.64 | 2.31-5.75 | <0.001* |
| NIV | 59 | 3.88 | 2.41-6.24 | <0.001* | 10 | 6.00 | 1.98-18.19 | 0.002* | 92 | 2.22 | 1.34-3.67 | 0.002* |
| Remdesivir  (at least 1 day prior to lab collection) | 2,293 |  |  |  | 820 |  |  |  | 1,452 |  |  |  |
| No | 2,268 | 1.00 | (reference) |  | 813 | 1.00 | (reference) |  | 1,438 | 1.00 | (reference) |  |
| Yes | 25 | 0.86 | 0.35-2.11 | 0.735 | 7 | 1.33 | 0.41-4.34 | 0.631 | 14 | 1.28 | 0.35-4.67 | 0.705 |
| AST | 2,171 |  |  |  | 773 |  |  |  | 1,342 |  |  |  |
| Normal | 961 | 1.00 | (reference) |  | 274 | 1.00 | (reference) |  | 789 | 1.00 | (reference) |  |
| Mild | 1,054 | 1.82 | 1.49-2.21 | <0.001* | 399 | 1.72 | 1.23-2.40 | 0.001* | 445 | 1.77 | 1.29-2.44 | <0.001* |
| Moderate | 139 | 1.98 | 1.43-2.73 | <0.001* | 90 | 2.36 | 1.57-3.54 | <0.001* | 92 | 2.47 | 1.55-3.92 | <0.001* |
| Severe | 17 | 2.69 | 1.39-5.23 | 0.003* | 10 | 2.06 | 0.76-5.53 | 0.154 | 16 | 2.24 | 0.79-6.38 | 0.131 |
| ALT | 2,174 |  |  |  | 776 |  |  |  | 1,347 |  |  |  |
| Normal | 1,353 | 1.00 | (reference) |  | 410 | 1.00 | (reference) |  | 987 | 1.00 | (reference) |  |
| Mild | 696 | 1.06 | 0.88-1.28 | 0.516 | 302 | 1.52 | 1.15-2.01 | 0.003* | 291 | 1.15 | 0.81-1.64 | 0.435 |
| Moderate | 119 | 0.98 | 0.66-1.45 | 0.908 | 59 | 1.81 | 1.18-2.77 | 0.006* | 57 | 1.31 | 0.67-2.55 | 0.432 |
| Severe | 6 | 0.88 | 0.15-5.29 | 0.890 | 5 | 2E^-6^ | 7E^-7^-5E^-6^ | <0.001* | 12 | 3.10 | 1.37-7.05 | 0.007* |
| AP | 2,172 |  |  |  | 776 |  |  |  | 1,348 |  |  |  |
| Normal | 1,767 | 1.00 | (reference) |  | 646 | 1.00 | (reference) |  | 1,007 | 1.00 | (reference) |  |
| Elevated | 405 | 1.13 | 0.91-1.39 | 0.268 | 130 | 1.34 | 0.98-1.83 | 0.063 | 341 | 1.74 | 1.28-2.35 | <0.001* |
| Total bilirubin | 2,172 |  |  |  | 777 |  |  |  | 1,348 |  |  |  |
| Normal | 1,980 | 1.00 | (reference) |  | 719 | 1.00 | (reference) |  | 1,139 | 1.00 | (reference) |  |
| Elevated | 192 | 0.91 | 0.66-1.25 | 0.564 | 58 | 1.43 | 0.95-2.15 | 0.086 | 209 | 1.98 | 1.43-2.74 | <0.001* |
| Direct bilirubin | 188 |  |  |  | 57 |  |  |  | 202 |  |  |  |
| Normal | 21 | 1.00 | (reference) |  | 3 | 1.00 | (reference) |  | 10 | 1.00 | (reference) |  |
| Elevated | 167 | 4.28 | 0.61-29.79 | 0.142 | 54 | 0.94 | 0.18-4.98 | 0.946 | 192 | 2.08 | 0.32-13.71 | 0.445 |
| GGT | 18 |  |  |  | 4 |  |  |  | 20 |  |  |  |
| Normal | 8 | 1.00 | (reference) |  | 0 |  | (reference) |  | 5 | 1.00 | (reference) |  |
| Elevated | 10 | 0.27 | 0.03-2.23 | 0.222 | 4 |  | unable to calculate |  | 15 | 7691500 | 1974262-29965205 | <0.001* |
| Albumin | 2,174 | 0.56 | 0.49-0.64 | <0.001* | 777 | 0.57 | 0.46-0.70 | <0.001* | 1,348 | 0.42 | 0.34-0.51 | <0.001* |
| Platelets | 2,286 | 0.998 | 0.997-0.999 | <0.001* | 818 | 0.997 | 0.995-0.999 | <0.001* | 1,448 | 1.00 | 1.00-1.00 | 0.102 |
| Creatinine | 2,287 | 1.02 | 1.00-1.05 | 0.041* | 820 | 0.99 | 0.94-1.05 | 0.780 | 1,449 | 1.02 | 0.97-1.06 | 0.467 |
| CRP | 1,885 | 1.03 | 1.03-1.04 | <0.001* | 666 | 1.04 | 1.02-1.05 | <0.001* | 593 | 1.03 | 1.01-1.05 | 0.001* |
| D-dimer | 1,721 | 1.03 | 1.01-1.04 | <0.001* | 596 | 0.96 | 0.92-0.996 | 0.031* | 432 | 1.01 | 0.97-1.05 | 0.722 |
| Hemoglobin | 2,285 | 1.02 | 0.99-1.06 | 0.175 | 819 | 1.05 | 0.99-1.11 | 0.092 | 1,448 | 0.97 | 0.91-1.02 | 0.243 |
| INR | 525 | 1.06 | 0.97-1.15 | 0.178 | 144 | 1.13 | 0.83-1.53 | 0.438 | 415 | 1.32 | 1.03-1.71 | 0.031* |
| PT | 525 | 1.00 | 0.99-1.01 | 0.480 | 144 | 1.01 | 0.99-1.04 | 0.387 | 415 | 1.02 | 1.00-1.05 | 0.025* |
| BUN | 2,287 | 1.01 | 1.01-1.01 | <0.001* | 819 | 1.01 | 1.00-1.01 | 0.017* | 1,448 | 1.01 | 1.00-1.01 | <0.001* |
| LDH | 385 | 1.00 | 1.00-1.00 | <0.001* | 45 | 1.00 | 1.00-1.00 | <0.001* | 124 | 1.00 | 1.00-1.00 | 0.923 |
| FIB-4 | 2,169 |  |  |  | 771 |  |  |  | 1,342 |  |  |  |
| Low | 628 | 1.00 | (reference) |  | 242 | 1.00 | (reference) |  | 453 | 1.00 | (reference) |  |
| Indeterminate | 813 | 2.03 | 1.52-2.71 | <0.001* | 298 | 2.09 | 1.37-3.19 | 0.001* | 444 | 1.88 | 1.17-3.00 | 0.009* |
| High | 728 | 3.33 | 2.53-4.38 | <0.001* | 231 | 3.26 | 2.18-4.89 | <0.001* | 445 | 3.38 | 2.20-5.18 | <0.001* |
| NFS | 2,148 |  |  |  | 765 |  |  |  | 1,325 |  |  |  |
| Low | 505 | 1.00 | (reference) |  | 232 | 1.00 | (reference) |  | 396 | 1.00 | (reference) |  |
| Indeterminate | 825 | 1.60 | 1.17-2.20 | 0.004* | 292 | 2.49 | 1.59-3.90 | <0.001* | 478 | 1.85 | 1.19-2.88 | 0.007* |
| High | 818 | 3.22 | 2.40-4.31 | <0.001* | 241 | 3.63 | 2.35-5.61 | <0.001* | 451 | 2.36 | 1.54-3.63 | <0.001* |
| APRI | 2,169 |  |  |  | 771 |  |  |  | 1,342 |  |  |  |
| Low | 1,049 | 1.00 | (reference) |  | 325 | 1.00 | (reference) |  | 800 | 1.00 | (reference) |  |
| Intermediate | 657 | 1.64 | 1.33-2.02 | <0.001* | 252 | 1.88 | 1.33-2.65 | <0.001* | 291 | 1.68 | 1.17-2.42 | 0.005* |
| High | 463 | 1.95 | 1.58-2.42 | <0.001* | 194 | 2.51 | 1.79-3.52 | <0.001* | 251 | 2.19 | 1.55-3.10 | <0.001* |
| De Ritis ratio | 2,171 |  |  |  | 773 |  |  |  | 1,342 |  |  |  |
| <1 | 472 | 1.00 | (reference) |  | 189 | 1.00 | (reference) |  | 278 | 1.00 | (reference) |  |
| 1-2 | 1,271 | 1.91 | 1.43-2.56 | <0.001* | 428 | 1.20 | 0.85-1.70 | 0.307 | 732 | 1.28 | 0.81-2.01 | 0.292 |
| >2 | 428 | 2.85 | 2.10-3.87 | <0.001* | 156 | 1.52 | 1.03-2.25 | 0.035* | 332 | 2.21 | 1.39-3.51 | 0.001* |
| LFT pattern | 2,172 |  |  |  | 776 |  |  |  | 1,347 |  |  |  |
| Mixed | 478 | 1.00 | (reference) |  | 216 | 1.00 | (reference) |  | 158 | 1.00 | (reference) |  |
| Hepatocellular | 80 | 0.77 | 0.43-1.38 | 0.386 | 48 | 1.24 | 0.79-1.96 | 0.354 | 36 | 1.62 | 0.74-3.55 | 0.232 |
| Cholestatic | 1,614 | 1.13 | 0.91-1.40 | 0.284 | 512 | 0.73 | 0.55-0.96 | 0.027* | 1,153 | 0.92 | 0.59-1.45 | 0.729 |

An asterisk (*) indicates statistical significance (p < 0.05). Abbreviations: RR=relative risk; CI=confidence interval; BMI=body mass index; CV=cardiovascular; HTN=hypertension; DM=diabetes mellitus; CKD=chronic kidney disease; HIV=human immunodeficiency virus; LFNC=low-flow nasal cannula; HFNC=high-flow nasal cannula; NIV=non-invasive ventilation; AST=aspartate aminotransferase; ALT=alanine aminotransferase; AP=alkaline phosphatase; GGT=gamma-glutamyl transferase; CRP=C-reactive protein; BUN=blood urea nitrogen; LDH=lactate dehydrogenase; INR=international normalized ratio; PT=prothrombin time; FIB-4=fibrosis-4 index; APRI=AST to platelet ratio index; NFS=non-alcoholic fatty liver disease fibrosis score; LFT=liver function test

# Supplemental Figures

##
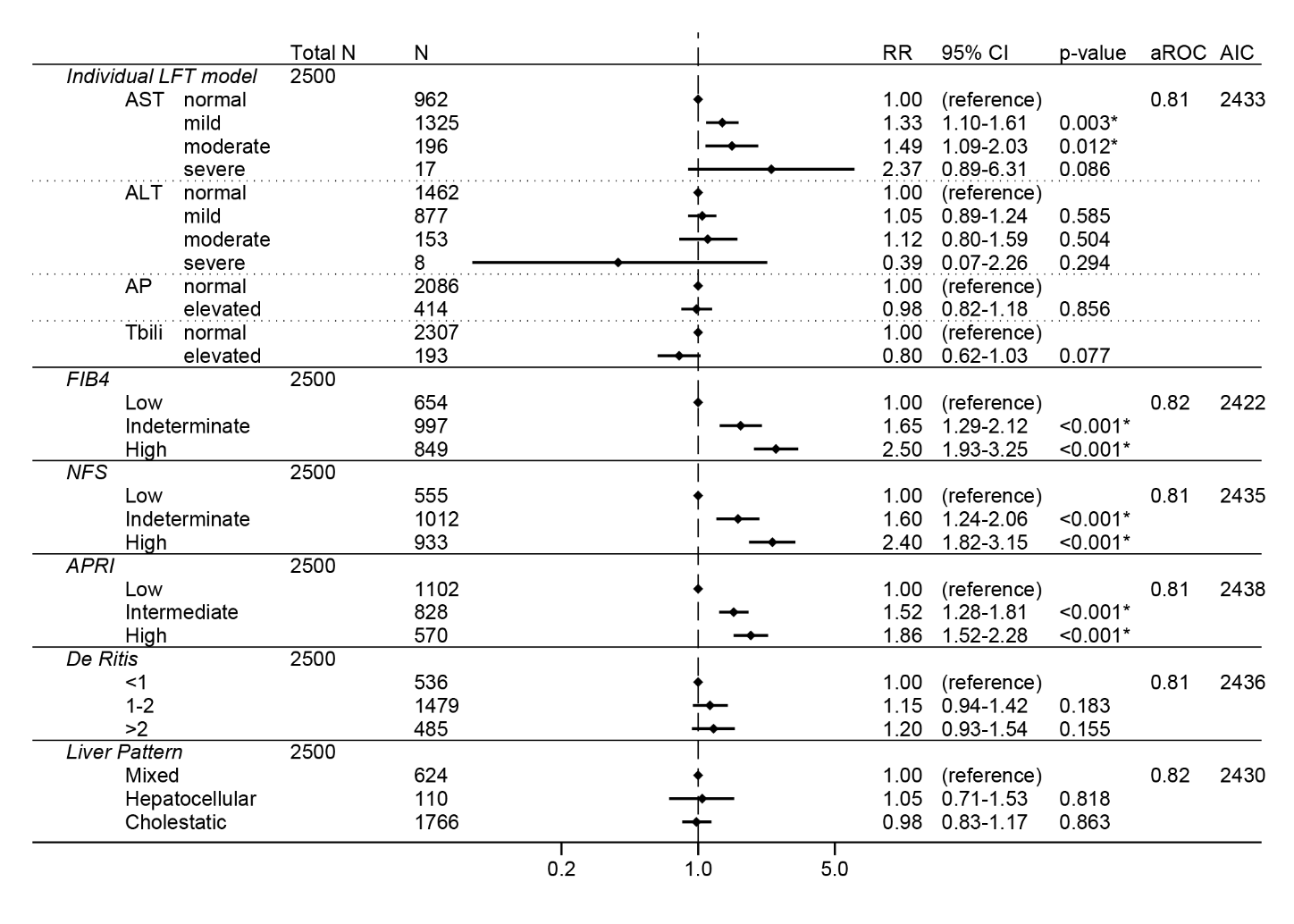
Figure S1. Adjusted relative risks of severe COVID-19 in the total cohort, including CRP and d-dimer in the models

Forest plots of relative risks of severe COVID-19 in the total cohort, including only patients with available CRP and d-dimer.

All models are adjusted for age, sex, race, ethnicity, body mass index, comorbidities (hypertension, cardiovascular complications, respiratory disease, diabetes mellitus, cancer, liver disease, viral hepatitis, HIV, rheumatologic disease, dementia, paralysis), baseline level of respiratory support, remdesivir prior to lab collection, creatinine, CRP, and d-dimer.

The individual LFT model was also adjusted for albumin and platelets.

The FIB-4 model was also adjusted for albumin, AP elevations, and total bilirubin elevations.

The NFS model was also adjusted for AP elevations and total bilirubin elevations.

The APRI model was also adjusted for ALT elevations, AP elevations, total bilirubin elevations, and albumin.

The De Ritis model was also adjusted for AP elevations, total bilirubin elevations, albumin, and platelets.

The liver pattern model was also adjusted for AST elevations, total bilirubin elevations, albumin, and platelets.

An asterisk (*) indicates statistical significance (p < 0.05).

Abbreviations: AST=aspartate aminotransferase, ALT=alanine aminotransferase, AP=alkaline phosphatase, Tbili=total bilirubin; FIB4=fibrosis-4 index; NFS=non-alcoholic fatty liver disease fibrosis score; APRI= AST to platelet ratio index; RR=relative risk; CI=confidence interval; aROC=area under the receiver operating characteristic curve; AIC=Akaike Information Criterion
